# Cell number in mesenchymal stem cell aggregates dictates cell stiffness and chondrogenesis

**DOI:** 10.1186/s13287-018-1103-y

**Published:** 2019-01-10

**Authors:** Melika Sarem, Oliver Otto, Simon Tanaka, V. Prasad Shastri

**Affiliations:** 1 Institute for Macromolecular Chemistry, University of Freiburg, Stefan-Meier Str.31, 79104 Freiburg, Germany; 2 BIOSS Centre for Biological Signaling Studies, University of Freiburg, 79104 Freiburg, Germany; 3 Helmholtz Virtual Institute on Multifunctional Biomaterials for Medicine, Kantstr. 55, 14513 Teltow, Germany; 4 Centre for Innovation Competence - Humoral Immune Response in Cardiovascular Diseases, University of Greifswald, Fleischmannstr. 42-44, 17489 Greifswald, Germany; 5 Computational Biology Group, D-BSSE, ETH Zürich, Mattenstrasse 26, 4058 Basel, Switzerland

**Keywords:** Mechanobiology, Caveolin-1, N-cadherin, Survivin, Matrix metalloproteinase-2, Developmental engineering

## Abstract

**Background:**

Although mesenchymal stem/stromal cell (MSC) chondrogenic differentiation has been thoroughly investigated, the rudiments for enhancing chondrogenesis have remained largely dependent on external cues. Focus to date has been on extrinsic variables such as soluble signals, culture conditions (bioreactors), and mechanical stimulation. However, the role of intrinsic mechanisms of MSC programming-based mechanobiology remains to be explored. Since aggregation of MSCs, a prerequisite for chondrogenesis, generates tension within the cell agglomerate, we inquired if the initial number of cells forming the aggregate (aggregate cell number (ACN)) can impact chondrogenesis.

**Methods:**

Aggregates of varying ACN were formed using well-established centrifugation approach. Progression of chondrogenic differentiation in the aggregates was assessed over 3 weeks in presence and absence of transforming growth factor-beta 1 (TGF-β1). Mechanical properties of the cells were characterized using high-throughput real-time deformability cytometry (RT-DC), and gene expression was analyzed using Affymetrix gene array. Expression of molecular markers linked to chondrogenesis was assessed using western blot and immunofluorescence.

**Results:**

Reducing ACN from 500 k to 70 k lead to activation and acceleration of the chondrogenic differentiation, independent of soluble chondro-inductive factors, which involves changes to β-catenin-dependent TCF/LEF transcriptional activity and expression of anti-apoptotic protein survivin. RT-DC analysis revealed that stiffness and size of cells within aggregates are modulated by ACN. A direct correlation between progression of chondrogenesis and emergence of stiffer cell phenotype was found. Affymetrix gene array analysis revealed a downregulation of genes associated with lipid synthesis and regulation, which could account for observed changes in cell stiffness. Immunofluorescence and western blot analysis revealed that increasing ACN upregulates the expression of lipid raft protein caveolin-1, a β-catenin binding partner, and downregulates the expression of N-cadherin. As a demonstration of the relevance of these findings in MSC-based strategies for skeletal repair, it is shown that implanting aggregates within collagenous matrix not only decreases the necessity for high cell numbers but also leads to marked improvement in the quality of the deposited tissue.

**Conclusions:**

This study presents a simple and donor-independent strategy to enhance the efficiency of MSC chondrogenic differentiation and identifies changes in cell mechanics coincident with MSC chondrogenesis with potential translational applications.

**Electronic supplementary material:**

The online version of this article (10.1186/s13287-018-1103-y) contains supplementary material, which is available to authorized users.

## Background

Self-aggregation of a dispersed cell population occurs during different stages of development such as embryogenesis, morphogenesis, and organogenesis, and the common notion is that it is due to intracellular adhesiveness and energy minimization [1–3]. A vital stage in endochondral ossification (EO) is condensation of mesenchymal stem/stromal cells (MSCs) [4–7], which starts with the formation of dense cell-cell contacts through adhesion proteins. The initiation of the process, size, boundaries, and differentiation of closely packed MSCs is tightly regulated via transmembrane adhesion proteins such as N-cadherin (N-cad) and N-CAM [8]. The cartilage matrix generated from differentiated MSCs lays the framework for the formation of long bones [9]. To date, efforts to enhance MSC chondrogenesis have focused on extrinsic variables such as soluble factors (transforming growth factor-beta 1(TGF-β1) [10], insulin-like growth factor (IGF), bone morphogenetic proteins (BMPs)) and optimizing the culture condition using different types of bioreactors including zero gravity and low-perfusion systems [11–13]. However, in vivo cells experience stresses (hydrodynamic, mechanical deformation) and this can manifest itself by changes to cell shape, cell volume, and membrane tension [14]. For example, it is well established that during development, cell fate and pattern formation is regulated by mechanical forces [15]. In this scenario, cell-cell contact plays an important role in transducing the mechanical forces into intra- and extracellular biochemical cues through activation of signaling pathways. For instance, it has been shown that mechanical stimulation can release latent stores of TGF-β1 from the extracellular matrix (ECM) [16–18]. Therefore, more recent mechanical stimulation of MSCs associated with a matrix (hydrogels, decellularized tissue) has emerged as another approach to enhanced chondrogenesis [19, 20]. In the aggregation step, MSCs are yet to secrete any extracellular matrix; therefore, the mechanical forces experienced by MSCs have to be derived intrinsically by the tension imposed by cell-cell contact during condensation [8]. Consequently, cell numbers within the MSCs aggregate represent an intrinsic variable whose impact on MSC chondrogenesis has remained unexplored. It is well appreciated that cell-cell adhesion involves cadherins, especially N-cad, which has been shown to play a vital role in MSC chondrogenesis [21, 22] and also has a mechanosensing function [23], and cytoplasmic anchoring of cadherins to the actin cytoskeleton is known to involve β-catenin [24], a transcriptional co-activator of MSC proliferation and differentiation [25]. Therefore, we theorized that the aggregation of MSCs could activate a hitherto unknown mechanobiology program for chondrogenesis involving modulation of N-cad and other potential players in mechanotransduction. In this study, we varied the number of human marrow-derived MSCs involved in the formation of aggregates (initial aggregate cell number (ACN)) and found that there is a direct correlation between ACN, mechanical properties of cells, and TCF/LEF-dependent transcriptional activity with MSC chondrogenesis could be modulated through an interplay between N-cad and Caveolin-1 (Cav-1), a protein residing in lipid rafts with a known role in mechanotransduction [26].

## Materials and methods

### Mesenchymal stem cell isolation and in vitro culture

Human marrow-derived mesenchymal stem cells (MSCs) were obtained from patients under informed consent in accordance to the regulations of the institution’s ethical committee (University Hospital Basel; ref. number of the local ethical committee: 78/07). In this study, cells from three donors ranging in age from 25 to 50 years (one female and two males) were used. The multi-lineage differentiation (osteogenic, chondrogenic, and adipogenic) potential of MSCs from all donors was confirmed by differentiation assays prior to the commencement of the studies. MSCs were expanded for two passages with α-minimum essential medium (MEM)-based media containing 10% FBS and 5 ng/mL FGF. MSC chondrogenic differentiation was carried out in aggregate culture system [27]. Briefly, 500,000, 350,000, 250,000, 150,000, and 70,000 MSCs were suspended in 0.5 mL serum-free chondrogenic differentiation media in 1.5 mL conical polypropylene tubes (Sarstedt, Germany) and centrifuged at 800 rpm for 3 min to form aggregates. Each condition was established in at least five replicates using independent multiple donors (*n* = 4–5). Aggregates were then cultured in serum-free medium for chondrogenic differentiation containing high glucose Dublecco’s MEM (DMEM) with non-essential amino acids (NEAA) and glutamine supplemented with 1 mM sodium pyruvate (Gibco, Germany), 100 mM HEPES buffer (PAN-Biotech, Germany), 100 U/mL penicillin, 100 mg/mL streptomycin (Gibco, Germany), and Insulin-Transferrin-Selenium (PAN-Biotech, Germany) supplemented with 0.1 mM ascorbic acid 2-phosphate (Sigma, Germany), 10 ng/mL TGF β-1 (R&D Systems), and 10^−7^ M dexamethasone (Sigma, Germany) for 3 weeks. The medium was changed twice a week, and the conditioned medium was collected, divided, and stored at 4 °C in the refrigerator for fibronectin quantification with ELISA assay and − 20 °C for zymography analysis. Aggregates were characterized using microscopy, histology, immunohistochemistry, RT-CD, and Affymetrix gene array. The study was performed with at least four biological replicates in the case of each donor and for each condition at each time point, and every biological replicate was measured at least twice. The data acquired from 26-year-old male donor have been presented in the manuscript.

### Histology and immunofluorescent staining

Aggregates were fixed in 3.7% paraformaldehyde for 2 h at 4 °C in the refrigerator, washed with PBS, and soaked in 3% sucrose overnight at 4 °C in the refrigerator and then embedded in OCT and cryosectioned. The sections (8-μm thick) were stained for GAG with Safranin-O and Alcian Blue. Immunohistochemical staining for extracellular matrix molecules was carried out using the following antibodies: collagen type II (Iowa University), type X (Abcam), fibronectin (FN) (Abcam), N-cadherin (Abcam), Cav-1 (cell signaling), and β-catenin (Abcam). After washing the samples to remove mounting media, the sections were treated for enzymatic antigen retrieval for Col II and Col X. In the case of Cav-1 and FN trypsin antigen retrieval was done prior to staining, and for N-cadherin and β-catenin, visualization heat antigen retrieval was used. Negative controls were established for each antibody by omitting the primary antibodies and using isotype control. For immunofluorescent (IF) visualization after incubation with primary antibodies, samples were incubated with secondary antibodies labeled with Alexa Fluor 594 and 488 (Invitrogen) followed by DAPI nuclear staining.

### Bern score

The Bern score assignment was performed as described by Grogan et al. [28]. Briefly, the evaluation of each aggregate was broken into three categories, and each category had four components with a score ranging from 0 to 3. A minimum Bern score is 0, and a maximum is 9. Samples were evaluated and scored by three independent observers.

**Table:** 

Scoring category	Score
A. Uniformity and darkness of Alcian Blue and Nuclear Fast Red staining	
No stain	0
Weak staining of poorly formed matrix	1
Moderately even staining	2
Even dark stain	3
B. Distance between cells/amount of matrix produced	
High cell densities with no matrix in between	0
High cell densities with little matrix in between	1
Moderate cell density with little matrix	2
Low cell density with moderate distance between cells and an extensive matrix	3
C. Cell morphologies represented	
Condensed/necrotic/pycnotic bodies	0
Spindle/fibrous	1
Mixed spindle/fibrous with rounded chondrogenic morphology	2
Majority rounded/chondrogenic	3

### Affymetrix gene array analysis

The total RNA was extracted from three biological samples for each condition using RNeasy Mini Kit (Qiagen kit) as recommended by the manufacturer. The quality and the quantity of isolated RNA were assessed using the NanoDropTM 2000C (Thermo Fisher Scientific). The quality and purity of RNA were further ascertained by ATLAS Biolabs (http://www.atlas-biolabs.com). The cDNA synthesis, amplification, purification, fragmentation and labeling, array hybridization, and scanning were done by ATLAS Biolabs according to the Affymetrix GeneChip™ technology. ATLAS Biolabs performed the microarray gene expression data analysis. The data have been processed using the Affymetrix Power Tools and Bioconductor R. The analyses have been performed using an RMA style background adjustment and quantile normalization (rma-sketch). Intensities are in log2 space. Detection above background (DABG) values was taken into account to filter for signals that are at least present in one group. A significance level of 5% and minimum twofold change cutoff were considered in the analysis.

### Modeling

The spherical aggregate was modeled as a sphere with isotropic continuous material properties with diffusion coefficient *D* and uptake rate *k*. The ambient compound concentration was set as a Dirichlet boundary condition for the domain partial differential equation (PDE). Discretization refinement studies were performed until convergence was achieved. To solve the PDEs, COMSOL Multiphysics 5.2a was used.

### Real-time deformability cytometry

Mechanical properties of cells are characterized by real-time deformability cytometry (RT-DC) as described elsewhere [29] using the Accelerator (Zellmechanik Dresden, Germany), a device that utilizes a 30 μm × 30 μm cross-section constriction within a microfluidic chip to measure the hydrodynamic deformation of cells. Cell size and deformation were quantified using high-speed image analysis of up to 1000 cells per second in real time. Prior to the experiment, cells were detached by treatment with trypsin for 5 min or via incubation with 0.3% type II collagenase (Invitrogen) for 1 h at 37 °C in an orbital shaker (in case of aggregates). Cells are centrifuged at 140×*g* for 5 min and resuspended in 0.6% (*w*/*v*) methylcellulose in Mg^2+^- and Ca^2+^-free PBS. The prefilled microfluidic chip was flushed with the cell suspension, and the flow was stabilized at 0.16 μl/s for 1 min before measurements. For each condition, approximately 1000 mechanical single cell measurements were acquired. Data analysis was carried out based on experimental duplicates from different days using linear mixed models [30, 31]. Briefly, by performing real-time deformability cytometry (RT-DC) on biological replicates each consisting of several hundreds of cells, the application of linear mixed models allows for separation of random and fixed effects in these large datasets. Fixed effects represent the quantity of interest while random effects summarize all systematic and random measurement bias. Practically, two models are stated, one with and one without the fixed-effect term, and statistical significance is calculated using Wilks theorem.

### Western blot analysis

Aggregates were lysed in Ripa buffer, and protein concentration was determined using BCA protein assay kit (Pierce). Twenty mircograms of protein was boiled with Laemmli buffer for 5 min at 95 °C and loaded in 8% (*w*/*v*) polyacrylamide-SDS gel and transferred it onto nitrocellulose membranes and blocked with 5% (*w*/*v*) BSA for 1 h at room temperature. The membranes were then probed with an antibody against caveolin-1 (Cell Signaling), β-catenin (Abcam), and FN (Technoclone); N-cadherin (Abcam) and GAPDH (Santa Cruz Biotechnology) were used as a loading control. Blots were developed using peroxidase-conjugated secondary antibodies and chemiluminescence system (Thermo Scientific). The intensities of bands were documented by a digital gel-imaging system (Thermo Scientific), and bands were analyzed by densitometry using ImageJ.

### Lentiviral production and transduction

Lentiviral particles containing shRNA constructs (7TGC lentiviral plasmid (Wnt reporter system with GFP and mCherry expression), Addgene plasmid 24304) and GIPZ Lentiviral shRNA clone: RHS4439 Open Biosystems, Germany, were produced in HEK293 cells, by co-transfecting lentiviral vector and packaging vectors using polyethylenimine (Mw 25.000, Sigma, Germany) as the transfection reagent. For transfection, 30 μg of DNA (4:3:1 of transfer vector, packaging coding vector (pCMVdR8.74) and envelope coding vector (pMD2.G)) was diluted in 250 μl Opti-MEM (Invitrogen, Germany) and 11.25 μL of polyethylenimine (1 mg/mL) was added to the solution, and the resulting mixture was incubated for 25 min at room temperature prior to adding to HEK293 cells. The medium was changed after 16 h to MSC expansion medium, and 64 h after transfection, the viral supernatants were collected and filtered through a sterile 0.45-μm syringe filter (Millipore, Germany). Viral particles were added to target cells (MSC). Three days after transduction, infected cells were selected by flow cytometry sorting for 7TGC vector and by adding 1 μg/mL puromycin (Sigma, Germany) to the culture medium in case of GIPZ.

### Flow cytometry

Cells within aggregates were digested via incubation with 0.3% type II collagenase (Invitrogen) for 1 h at 37 °C in an orbital shaker. Harvested cells were centrifuged and suspended in flow cytometry buffer (PBS containing 2% FBS). A total of 10,000 events were recorded for each sample using flow cytometry (Gallios; Beckman Coulter). Samples were analyzed with Flowing software, and an average of at least five different samples was calculated.

### Gelatin zymography

Polyacrylamide/sodium dodecyl sulfate (SDS) gel electrophoresis (Page) was performed using 10% gels containing 1 mg/mL of gelatin (Sigma, Germany). SDS was removed by washing in 2.5% Triton X-100 for 1 h at room temperature before the enzyme reaction. The gel was incubated overnight at 37 °C in enzyme buffer containing 50 mmol/L Tris, pH 7.5, 200 mmol/L NaCl, 5 mmol/L CaCl2, and 0.02% Brij-35. The MMP-2 activity was identified by the area where the gelatin was degraded which appears as a distinct white band after staining the gel with simply blue (Invitrogen, Germany) for 2 h at room temperature followed by overnight washing with water. The imaging and analysis were done using the same methodology as for western blot analysis.

### Fibronectin ELISA

A sandwich ELISA for the detection of soluble FN was developed in our laboratory. Briefly, 96-well ELISA plates were coated with mouse anti-human monoclonal FN antibody (mAb 6 FN, Technoclone, Austria) and incubated at 4 °C overnight. The plates were then washed with PBS (0.05% Tween 20) and blocked overnight in the fridge. Conditioned media (100 μL) from different samples were added to the wells in triplicate and incubated for 2 h at room temperature. Plates were then washed three times and incubated at room temperature with detection antibody (biotinylated rabbit polyclonal anti-FN (Abcam) for 2 h. The plates were then washed and incubated with UltraAvidin-Horseradish Peroxidase coupled secondary goat anti-rabbit (Abcam) for 30 min at room temperature, and the color was developed with one step substrate (R&D Systems) and the absorbance at 570 nm was measured using a plate reader (BioTek Synergy HT). The absorbance at 450 nm was subtracted from to yield the corrected values for analysis.

### Proliferation analysis

Human MSCs were stably transfected with ShRNA for green fluorescence protein, and aggregates were induced using different initial cell numbers. At pre-determined time points, the aggregates were dissociated with 0.3% collagenase at 37 °C and the cell numbers were obtained using a plate reader (BioTek Synergy HT). A calibration curve was established to relate fluorescence intensity to cell numbers, and this was used to determine the cell number in the digest.

### In vitro culture using collagenous matrix

One million MSCs were cultured in type I collagen meshes (Ultrafoam®, Davol, Warwick, RI, USA) with 4-mm diameter, and 4 pellets of 70 k were implanted in collagen meshes 2 days of incubation. Both conditions were cultured using serum-free medium for chondrogenic differentiation for 3 weeks. The engineered constructs were cryosectioned and histologically stained (Safranin-O staining) in order to analyze the quality of deposited tissue. The Bern score was assessed for different constructs by virtually dividing each construct to10 circular regions. Bern score was plotted against the distance of each circular region form center of constructs.

### Statistical analysis

All values are reported as mean ± standard deviation and *p* values greater than 0.05 were set as not significant (ns). Significance is notated with asterisks as follows: *p < 0.05, **p < 0.01, and ***p < 0.001. Statistical analysis (in all measurements except values extracted from RT-DC (see the “Real-time deformability cytometry” section for details)) was performed with OriginPro 2017. For statistics, a one-way analysis of variance (ANOVA) with post-hoc Tukey pairwise comparison was used.

## Results and discussion

### ACN activates and modulates early phase of chondrogenic differentiation in MSCs independent of exogenous soluble TGF-β1

**Fig. 1:**
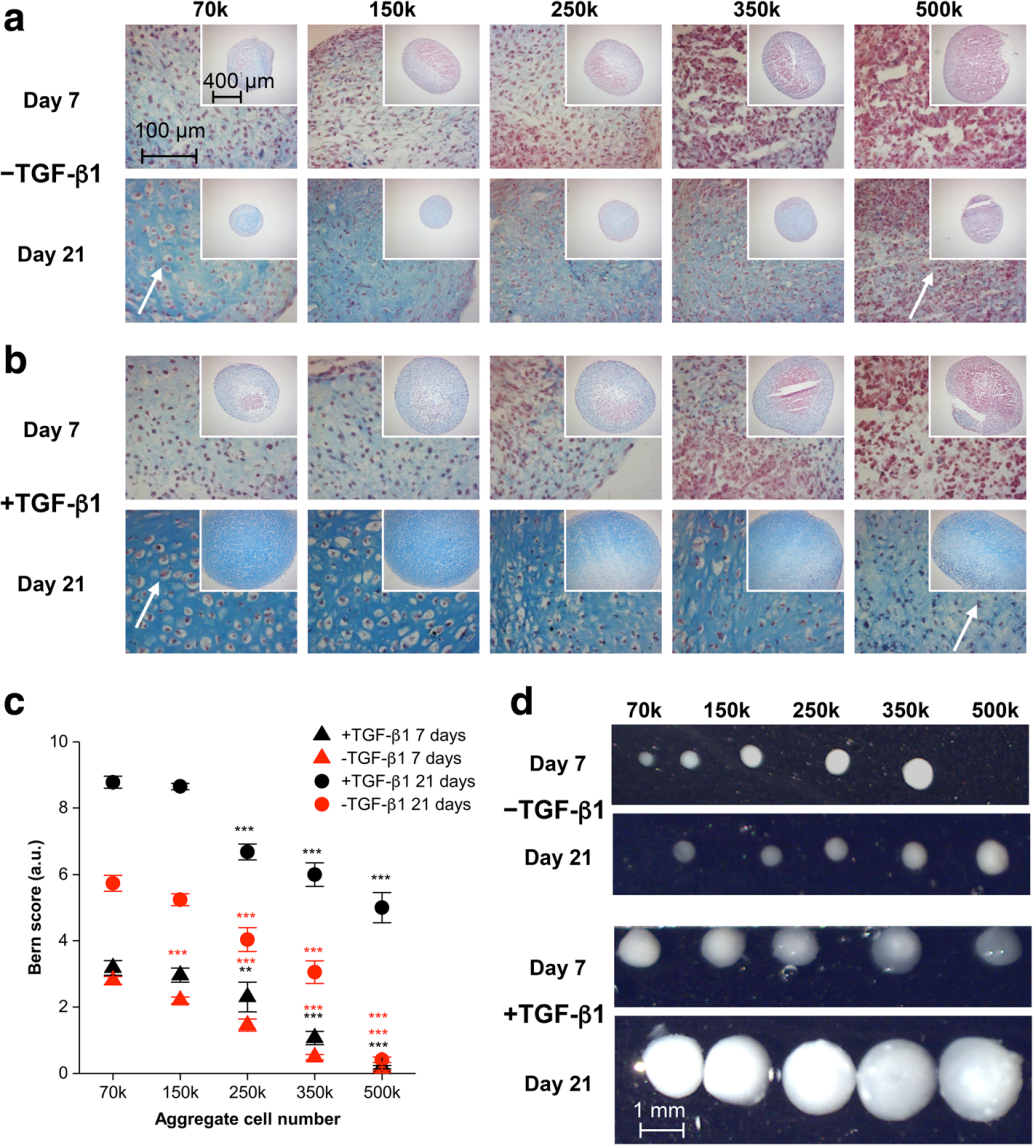
Initial cell number during aggregation regulates chondrogenic differentiation of human bone marrow-derived MSCs. Alcian Blue staining of pellet sections grown in the absence **a** and presence **b** of TGF-β1 after 7 and 21 days of differentiation. The insets show the low magnification image. **c** Bern score associated with each MSC aggregates differentiated in the presence and absence of TGF-β1 at different time points determined from Alcian Blue staining of pellets. Statistical analysis is based on a one-way ANOVA tool of OriginPro. Error bar represents standard deviation, and different aggregates in one group are compared to 70 k in the same group (*p < 0.05, **p < 0.01, ***p < 0.001). **d** Macroscopic images of pellets in the presence and absence of TGF-β1

Since it has been suggested that in limb mesenchyme prior to condensation contains endogenous TGF-β1 [32], we first inquired, if ACN by itself is capable of inducing the expression of glycosaminoglycans (GAGs), an early sign of chondrogenesis, even in the absence of exogenous TGF-β1. In order to ascertain this, we employed the well-established 3D aggregate culture system [27] and cultured MSCs aggregates with different ACNs (70 k, 150 k, 250 k, 350 k, and 500 k) for 7 and 21 days in the absence of TGF-β1, and we observed that the lowest ACNs of 70 k showed adequate staining of GAGs and ACN of 150 k showed mild staining for GAGs even at day 7. However, above this ACN, low to no GAGs expression was observed (Fig. 1a). In presence of TGF-β1, the expression of GAGs was enhanced as expected, but again it was most pronounced in ACN of 70 k and 150 k, and the trend of decreasing GAGs expression with increasing ACN was maintained (Fig. 1b). It is noteworthy that the expression of GAGs on day 7 was observed both in the absence and presence of TGF-β1 in ACN of 70 k, thus providing the first evidence that the activation of the chondrogenic program in MSCs upon condensation is independent of soluble TGF-β1 and it is an intrinsic mechanism. However, on day 21, the +TGF-β1 condition showed a substantial increase in the quantity (size of cartilaginous mass, Fig. 1b) and quality of the GAG matrix; however, the impact of ACN on the organization and shape of the cells and the uniformity and intensity of the GAG matrix was still evident (Fig. 1a, b; Additional file 1: Figure S1).

### Lower ACN enhances matrix production during MSC chondrogenesis in a TGF-β1-dependent manner

We then proceeded to quantify the quality of the cartilaginous matrix using the Bern score, an accepted semi-quantitative metric to describe chondrogenesis [28]. In this scoring system, three factors go into assessing the quality of the cartilage matrix: (1) cell morphology, (2) distance between cells/amount of matrix produced uniformity, and (3) intensity of Alcian Blue staining, and each factor is given a score ranging from 0 to 3. Thus, aggregates with highest chondrogenic differentiation have a score of 9, and the ones with the least have a score of 0. The histological analysis and Bern score assessment of aggregates at day 7 of chondrogenic differentiation revealed that addition of TGF-β1 does not alter the Bern score of aggregates significantly (Fig. 1c). In both conditions (± TGF-β1), cells within the *low*-ACN aggregates particularly in the periphery had already acquired chondrogenic morphology (rounded) and showed expression of GAGs with a Bern score in the range of ~ 3. In comparison, *high*-ACN conditions promoted a fibroblastic morphology and showed little-to-no GAG production throughout the aggregate cross-section and had a negligible Bern score. This is a significant observation as it suggests that in the early stages of MSCs-derived chondrogenesis, intrinsic signaling plays a dominant role. However, at day 21, although the clear trend of higher chondrogenesis at lower ACN was sustained, the Bern score was consistently higher as expected in presence of TGF-β1. This trend between ACN and GAGs expression was also confirmed in other donors (age 25–56 years old male and female) confirming the generality of the observations (Additional file 1: Figure S2).

Visual inspection of MSCs aggregates using light microscopy revealed that in presence of TGF-β1, despite the fact that the lowest ACN condition (70 k) (*low*-ACN) had seven times less cells at the onset compared to the highest ACN (500 k) (*high*-ACN), after 7 days, the volumetric size difference among aggregates was negligible, and after 21 days, the diameter of the *high*-ACN aggregates was only around 1.5 times of the *low*-ACN aggregates (Fig. 1d). However, such a dramatic change from day 7 to day 21 was not present in absence of TGF-β1, again confirming that ACN acts as a trigger for chondrogenic differentiation of MSCs; however, matrix production requires exogenous signals. This led us to postulate that either ECM production in aggregates with *low*-ACN was more efficient, and/or cells within this environment were able to undergo proliferation. Since the effect of ACN was preserved even in presence of TGF-β1, all further analysis was undertaken in presence of TGF-β1.

### ACN impacts the expression of genes regulating chondrogenesis and endochondral ossification

**Fig. 2:**
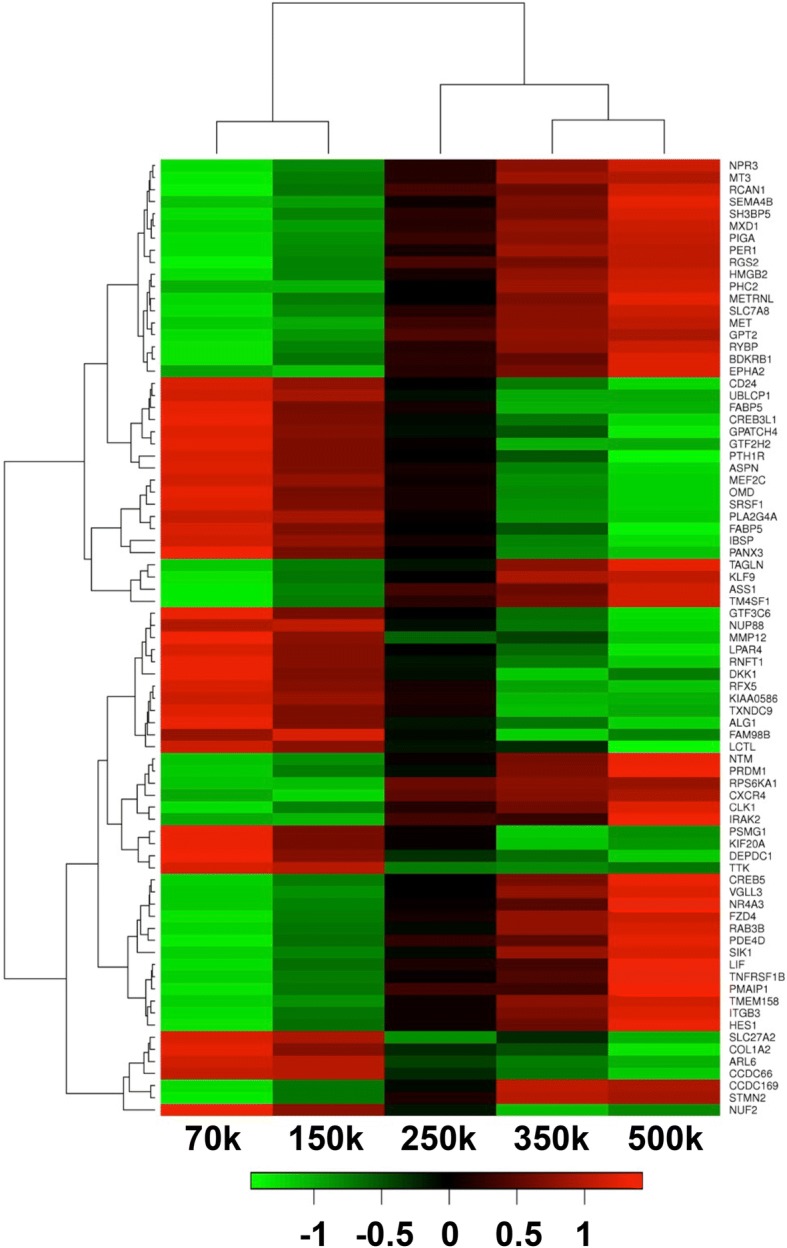
Affymetrix gene array analysis of pellets at day 2 of chondrogenic differentiation in the presence of TGF-β1. The genes associated with chondrogenesis and endochondral ossification are upregulated in pellets with lower initial cell number

To investigate the impact of ACN on gene expression pattern in MSCs, we employed Affymetrix gene array analysis. Cells were isolated from pellets formed in presence of TGF-β1 48 h past initiation of chondrogenic differentiation. A heat map showing the top 100 differentially regulated genes as a function of initial ACN is shown in Fig. 2. Noteworthy, genes playing a very important role in chondrogenesis such as parathyroid hormone-1 receptor (PTH1R) [33] was upregulated by more than sixfold in 70 k pellets. Interestingly, genes regulating Wnt/β-catenin signaling cascade such as frizzled class receptor 4 (FZD4) and dickkopf-related protein 1 (DKK1) were significantly (more than threefold) altered in low ACNs. Based on this information, we postulate that the Wnt/β-catenin signaling pathway could be one of the main modulators of ACN-induced chondrogenesis. Additionally, genes such as integrin-binding sialoprotein (IBSP), alkaline phosphatase (ALPL), bone morphogenetic protein 4 (BMP4), pannexin 3 (PANX3), asporin (ASPN), Indian hedgehog (IHH), and hes family bHLH transcription factor 1 (HES1) with a role in development of cartilage and EO were highly upregulated in low ACNs. Interestingly, genes associated with cellular response to lipids (GO:0071396) such as bradykinin receptor B1 (BDKRB1), argininosuccinate synthase 1 (ASS1), high mobility group box 2 (HMGB2), interleukin-1 receptor-associated kinase 2 (IRAK2), phosphodiesterase 4D (PDE4D), Kruppel-like factor 9 (KLF9), nuclear receptor subfamily 4, group A, member 3 (NR4A3), and tumor necrosis factor receptor superfamily, member 1B (TNFRSF1B) were all highly downregulated in *low*-ACNs.

Upon analysis of the top 300 differentially regulated genes (min 2.2-fold), we found out that genes involved in regulation of lipid storage (GO:0010883) such as caveolin-1 (CAV1), interleukin 6 (IL6), CD36, nuclear factor of kappa light polypeptide gene enhancer in B cells inhibitor (NFKBIA), and integrin beta 3 (ITGB3) and regulation of lipid biosynthetic process (GO:0046890) and regulation of lipid transport (GO:0032368) such as nuclear receptor subfamily 1, group D, member 1 (NR1D1), leptin (LEP), complement component 3 (C3), and salt-inducible kinase 1 (SIK1) were all downregulated in *low*-ACNs.

Additionally, we did not see significant differences in expression of hypoxia-associated genes such as hypoxia-inducible factors 1 and 3 (HIF1A, HIF3A), egl-9 family hypoxia-inducible factor 3 (EGLN3) [34], and hypoxia-inducible lipid droplet-associated (HILPDA) between MSCs from various ACNs. This was an interesting observation as it has been widely accepted that multicellular tumor spheroids experience hypoxia [35, 36] and the diffusion of nutrition and oxygen in multicellular aggregates is compromised in direct relation to the aggregates size [37].

### Modeling of oxygen and TGF-β1 diffusion and glucose consumption in different ACNs

Oxygen availability has been frequently suggested as one of the primary factors influencing MSC fate [38]. It has been reported that differentiating embryoid bodies experience gradients of nutrients, oxygen, and cytokines and that the concentration of oxygen at the centers of embryoid bodies with a radius of 400 μm was 50% lower than that in embryoid bodies with a radius of 200 μm [39]. Even though in this study aggregates at day 7 of differentiation have a relatively similar size (Additional file 1: Table S1), in order to rule out the impact of oxygen and nutrient diffusion in the observed outcomes, we modeled the diffusion of oxygen, glucose, and TGF-β1 in aggregates of varying ACNs at day 7 of chondrogenic differentiation.

The diffusion of the molecules is modeled as Fick’s law of diffusion, described by the following partial differential equation (PDE):

$$ \frac{\partial c}{\partial t}-D\Delta  c+ kc=0 $$

where *c* = *c* (*x*, *t*) is the compound concentration at location *x* and time *t*, *D* is the diffusion coefficient, *∆* is the Laplace operator, and *k* is the uptake rate. The data described in Additional file 1: Table S2 was used.

**Fig. 3:**
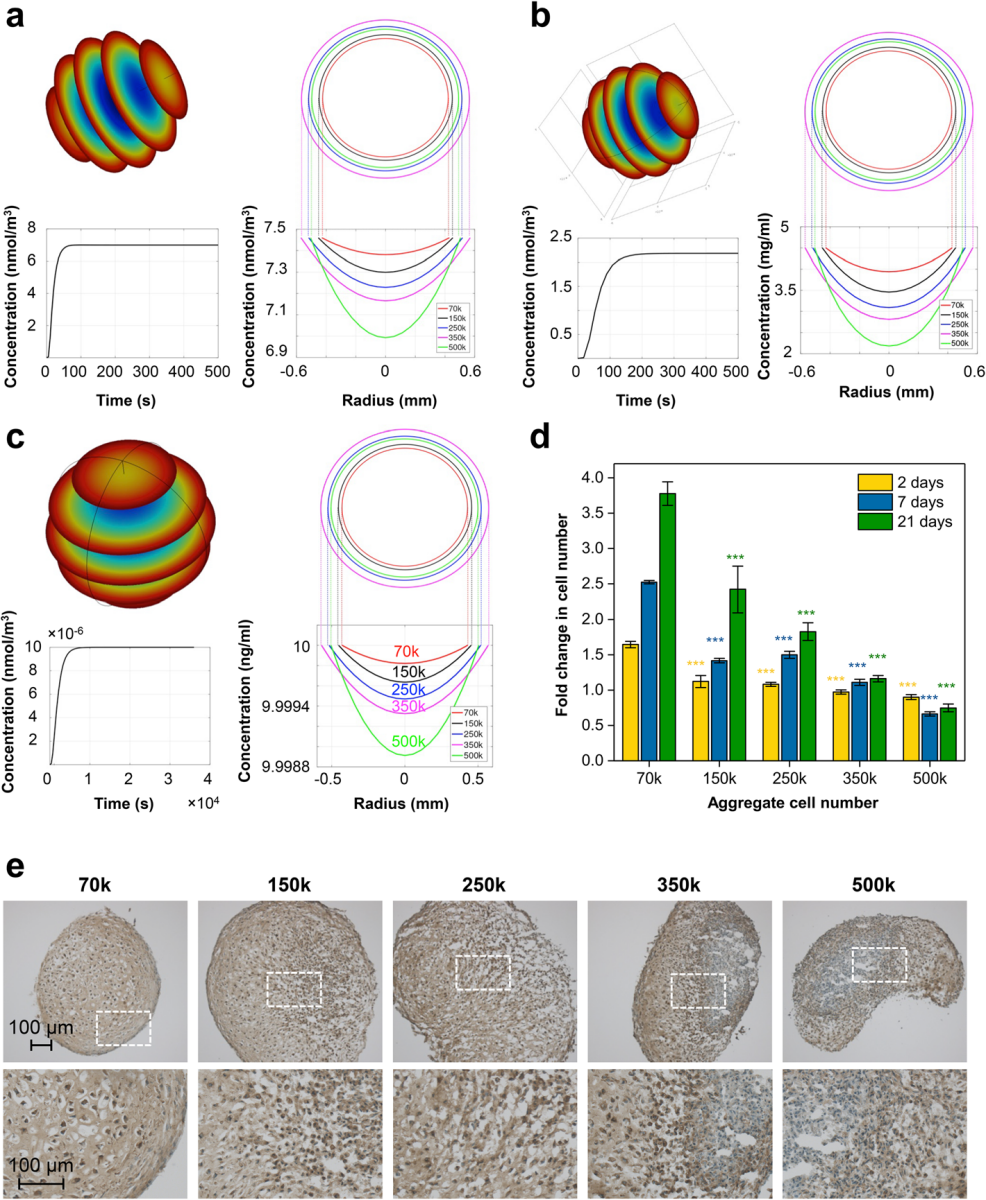
Initial cell number does not impact oxygen and nutrition diffusion. Mathematical modeling for oxygen diffusion (**a**), glucose consumption (**b**), and TGF-b1 concentration (**c**) within pellets at day 7 of chondrogenic differentiation. Top left in **a**–**c**: heatmap on slices through the spherical cell pellet at steady state (red, high concentration; blue, low concentration). Top right in **a**–**c**: size comparison of the spherical aggregates for pellets with different initial cell number. Bottom left in **a**–**c**: temporal concentration development in the center of a 500 k sphere. Steady state is reached within minutes; hence, a steady state analysis is justified. Bottom right in **a**–**c**: concentration profiles across the spherical aggregates at steady state. **d** Ratio of cell number within the pellets normalized to initial cell number at different time points. Cells within pellets with lower initial cell number show higher proliferation potential, which is in agreement with the higher concentration of glucose within low-ACN pellets. Statistical analysis is based on a one-way ANOVA tool of OriginPro. Error bar represents standard deviation and different aggregates in one group are compared to 70 k in the same group (*p < 0.05, **p < 0.01, ***p < 0.001). (**e**) HIF-1α expression in pellets at day 7 of chondrogenic differentiation confirms the modeling data that cells in all conditions experience similar oxygen tension

As per modeling, the oxygen concentration at the center of the pellet is expected to be similar to the edge of the pellet with a less than 10% difference observed in the case of the 500 k pellets (Fig. 3a). The differences in oxygen concentration predicted for various ACNs do not constitute hypoxia as even larger variations are observed in human physiology in the range of 3–7% oxygen [40]. Likewise, the drop in the concentration of TGF-β1 is also predicted to be negligible (Fig. 3c). However, the glucose concentration at the center of the 500 k pellets is predicted to be half (~ 2.2 mg/mL) of that in media (4.5 mg/mL), with no such drastic differences at the center of the *low*-ACN pellets (Fig. 3b). Since glucose is a small molecule, the only factor that can alter its diffusion behavior is consumption rate. Therefore, a study to examine the proliferation status of the MSCs within different pellets was undertaken. Aggregates were formed using MSCs stably expressing Turbo-GFP and cell numbers were quantified at days 2, 7, and 21 after aggregate formation. Surprisingly, cell proliferation showed an inverse correlation with an increase in ACN, with cell numbers by day 21 within *low*-ACN aggregates being fourfold higher (after normalization to initial cell number) in comparison with *high*-ACN (Fig. 3d). This correlates with the lower glucose concentration predicted at the interior of 500 k and 350 k aggregates. This observation is quite significant as we have shown that cartilage engineered de novo in skeletally mature rabbits by inducing chondrogenesis from periosteal progenitor cells is hypercellular and therefore capable of undergoing remodeling in vivo as they try to attain cellular homeostasis [41]. However, further experiments are necessary to understand the role of glucose consumption in *low*-ACNs aggregates in driving chondrogenesis. Aggregate formation has also been reported to induce the expression of hypoxia-inducible factor (HIF)-1α in particular in the interior. Immunostaining of cryosections of aggregates for HIF-1α revealed that while both low- and high-ACNs show global expression of HIF-1α, in aggregates formed from ACN of 350 k and 500 k an increase in expression of HIF-1α in the cells in close proximity of aggregate core is additionally observed (Fig. 3e). Nonetheless, the absence of any remarkable differences in the diffusion of oxygen and TGF-β1 in the various ACN scenarios pointed to possibly others mechanisms for the observed differences in the chondrogenic capacity of the MSCS.

### ACN programs MSCs biomechanics with increasing stiffness in cells correlating with higher chondrogenesis

**Fig. 4:**
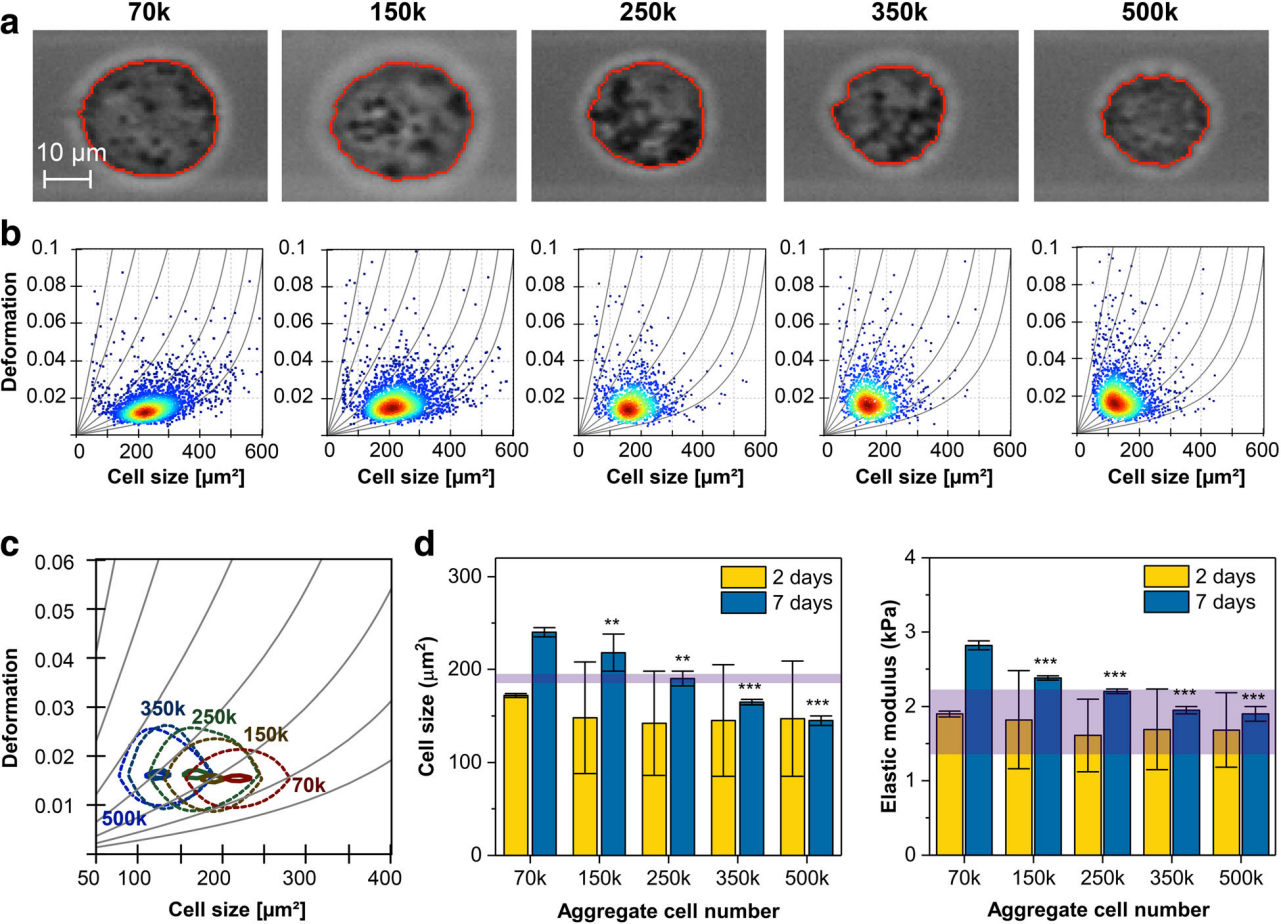
MSC stiffness correlates with chondrogenic potential and is influenced by cell number within aggregates. **a** Images of suspended cells in RT-DC at day 7 of chondrogenic differentiation for different ACNs. **b** RT-DC scatter plots of cell size and deformation at day 7 for different ACNs. Each scatter plot summarizes more than 1000 cells per condition. Isoelasticity lines in gray highlight areas of equal elastic Young’s modulus. Color code indicates red (maximum) to blue (minimum) cell density. **c** Contour plot showing 50% (dashed) and 90% (solid) of maximum event density for aggregates at day 7 in different conditions: 70 k (red), 150 k (yellow), 250 k (dark green), 350 k (dark blue), and 500 k (blue). **d** Statistical analysis comparing elastic Young’s modulus and cell size to 70 k ACN condition. For day 2, cells were pooled from three to four aggregates to establish a mean for a particular condition, and day 7 shows data from experimental replicates analyzed by linear mixed models. Error bars represent the standard deviation of the distribution (day 2, one pooled sample) and standard error of the mean of the replicates (day 7, three pooled samples). The purple band represents the range of values for undifferentiated MSCs (*p < 0.05, **p < 0.01, ***p < 0.001)

There is strong evidence that the mechanical properties of cells can contribute to fate choices [42]. The mechanical property of the cell is determined by different cellular components such as plasma membrane, cytoskeleton, and organelles [43, 44]. It has been suggested that the presence of cholesterol and poly-saturated fatty acids in the lipid bilayer increases the stiffness [45]. We have recently shown that cell plasma membrane lipid bilayer composition is altered by lipid transfer, and this impacts the deformability of cell membrane [46]. Since several genes associated with lipid transfer and lipid storage were downregulated in *low*-ACNs, we investigated if these differences at the gene expression level translate into changes in the stiffness of cells. Single cells dissociated from the aggregates were subjected to real-time deformability cytometry (RT-DC) and characterized for size and deformation at days 2 and 7 of chondrogenic differentiation [29]. Qualitatively, we observed a decrease in projected cell size for increasing ACN at day 7 (Fig. 4a) while the size deformation distribution of more than 1000 single cell measurements per condition shows little variation in deformation; the trend of decreasing cell size by increasing ACN was evident (Fig. 4b). A direct comparison using the probability density function and applying an analytical model to extract mechanical properties [47] suggests that 70 k aggregates have a higher elastic modulus than their counterparts, i.e., *high-*ACN aggregates (Fig. 4c). Additionally, our analyses suggest that at an earlier stage of chondrogenesis (2 days), varying ACN does not significantly impact the cell size. However, after 7 days, cells from *low*-ACNs were significantly larger, which interestingly correlates with their higher chondrogenic potential (Fig. 4d, left panel). Purple band indicates values from undifferentiated MSCs from 2D culture. Analysis of the elastic Young’s modulus (*E*) revealed no clear differences between cells from the various ACN conditions on day 2. However, at day 7, cells of 70 k ACN, which are more chondrogenic, were also significantly stiffer than cells from higher ACNs, which show diminished chondrogenicity (Fig. 4d, right panel). Interestingly, the qualitative comparison between Young’s modulus (*E*) of cells from *high*-ACNs (250 k and greater) showed similar values to *E* of undifferentiated MSCs (purple band across graph). This is the first report to link chondrogenesis to an emerging mechano-phenotype in MSCs.

### Expression of mechanosensing proteins N-cadherin and caveolin-1 in MSCs aggregates is modulated by ACN

In order to ascertain the mechanism underpinning the regulation of chondrogenesis by ACN, we investigated the expression of proteins involved cell-cell contact. One of the proteins known to inhibit cell-cell contact in epithelial cells is Cav-1 [48]. Cav-1 is the main scaffolding protein residing in the cholesterol-rich membrane micro-domains (caveolae), which has a documented role in mechanotransduction in endothelial cells [49] and also implicated in transduction of mechanical forces across cell-cell junctions via stretch-activated channels [50]. Caveolae have been implicated in the compartmentalization and regulation of many signaling events such as MSC renewal and differentiation (adipogenic and osteogenic) [26], and its expression has been observed during chondrogenesis in the tibiotarsus (avian limb) and in chondrocytes in the vicinity of the proliferating zone within the cartilage [51] Furthermore, Cav-1 knockout mice show an increase in length of growth plate, number of hypertrophic cells, bone size, and stiffness [52, 53]. Notwithstanding, the relevance of Cav-1 in MSC condensation and chondrogenesis remains ill-defined.

**Fig. 5:**
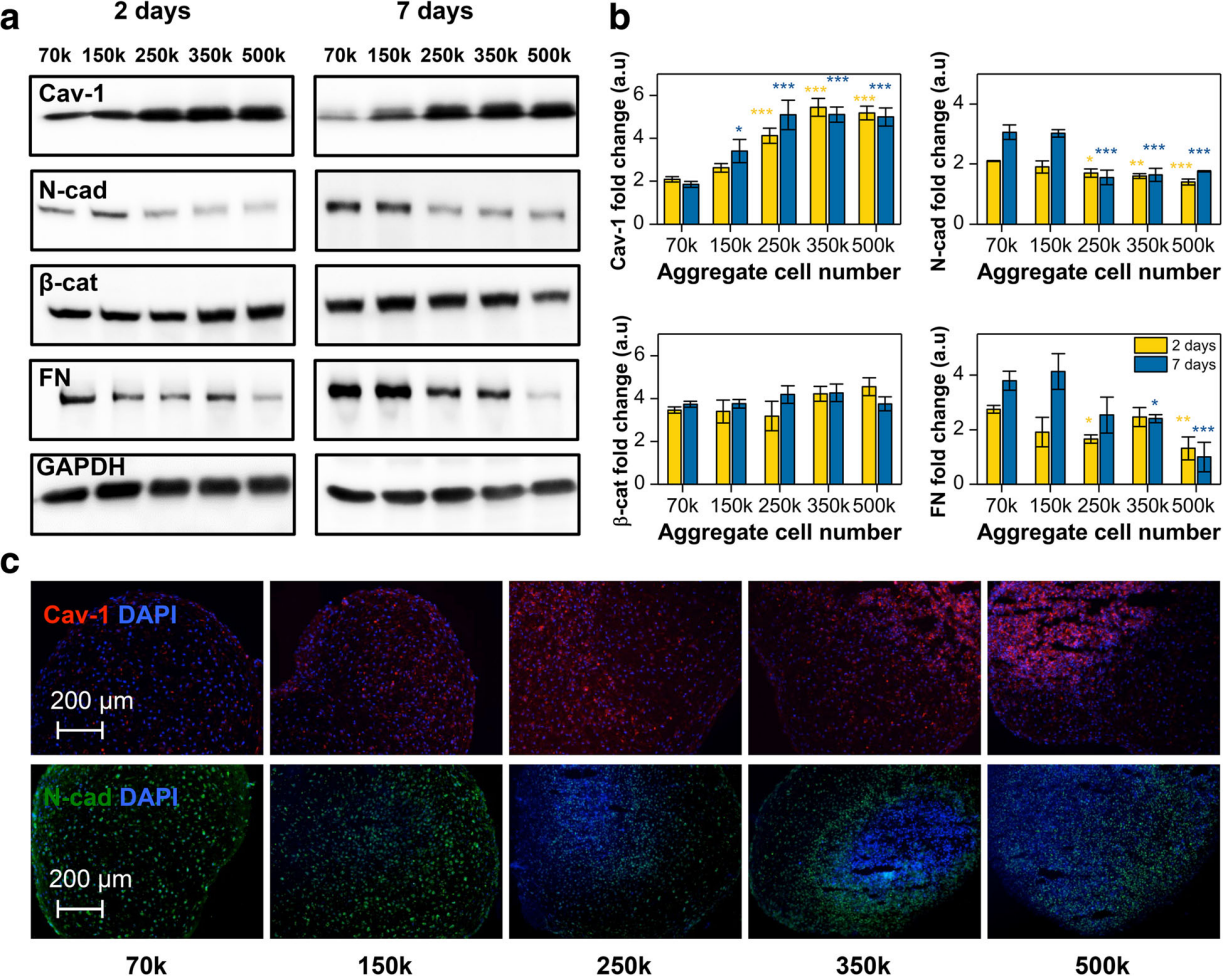
Initial cell number modulates the expression of different mechanosensing proteins. **a** Western blot analysis revealed that initial cell number significantly impacts the expression of Cav-1, N-cad, β-cat, and FN. **b** Quantification of western blot analysis data. Values are normalized to GAPDH. Statistical analysis is based on a one-way ANOVA tool of OriginPro. Error bars are standard error of the mean, and different aggregates in one group are compared to 70 k in the same group (*p < 0.05, **p < 0.01, ***p < 0.001). **c** Immunofluorescence staining for Cav-1 and N-cad in sections of pellets after 7 days of differentiation

Western blot (WB) analysis revealed that as early as 2 days after induction of differentiation Cav-1 expression showed an unambiguous and direct correlation with ACN, with the *high*-ACN aggregates having the most pronounced expression which after 7 days of differentiation was 4–5-fold higher compared to *low*-ACN aggregates (Fig. 5a). This is also in agreement with our Affymetrix gene array data, which showed downregulation of CAV1 by 2.4-folds in *low*-ACN. However, after 21 days, a general downregulation of Cav-1 in all conditions was observed with no appreciable differences (Additional file 1: Figure S4). In contrast, N-cad expression showed a completely opposite trend, with *low-*ACN conditions already showing appreciable expression by day 2 which after 7 days was 2–3-fold higher compared to *high-*ACN condition, implying that increasing ACNs during MSC aggregate formation has a negative effect on N-cad expression and stabilization (Fig. 5a). This overall trend was confirmed by IF staining that revealed a high punctuate expression of Cav-1 during the condensation phase in *high*-ACN aggregates and vice versa for *low*-ACN aggregates (Fig. 5b) and dramatic decrease in N-cad expression going from *low*- to *high*-ACNs, with the core of *high-*ACN aggregates almost devoid of N-cad expression (Fig. 5c). This positive correlation between N-cad expression and chondrogenesis in human MSCs is consistent with the findings in avian limb chondrogenesis where perturbation of N-cad function was found to inhibit cellular condensation and chondrogenesis [21, 22]. Interestingly, in murine skeletal myoblasts, the dynamic assembly of N-cad at cell-cell contact involves lipid rafts in particular caveolae [54]. Since it is accepted that Cav-1 has a role in contact inhibition where redistribution of Cav-1 from punctate region on the cell surface to nodes at cell-cell junctions occurs [48, 55], our finding that in human MSCs that N-cad and Cav-1 expression shows inverse relationship therefore alludes to a possible negative regulatory effect of Cav-1 on N-cad expression and stabilization, and a more general role for Cav-1/N-cad interplay in regulating cell function. Additionally, this interesting inverse relationship between two proteins implicated in mechanotransduction provides circumspect evidence for two opposing mechanobiology mechanisms responsible for directing chondrogenesis in MSCs.

### ACN influences TCF/LEF transcriptional activity and expression of N-cad and Cav-1 is MSCs

**Fig. 6:**
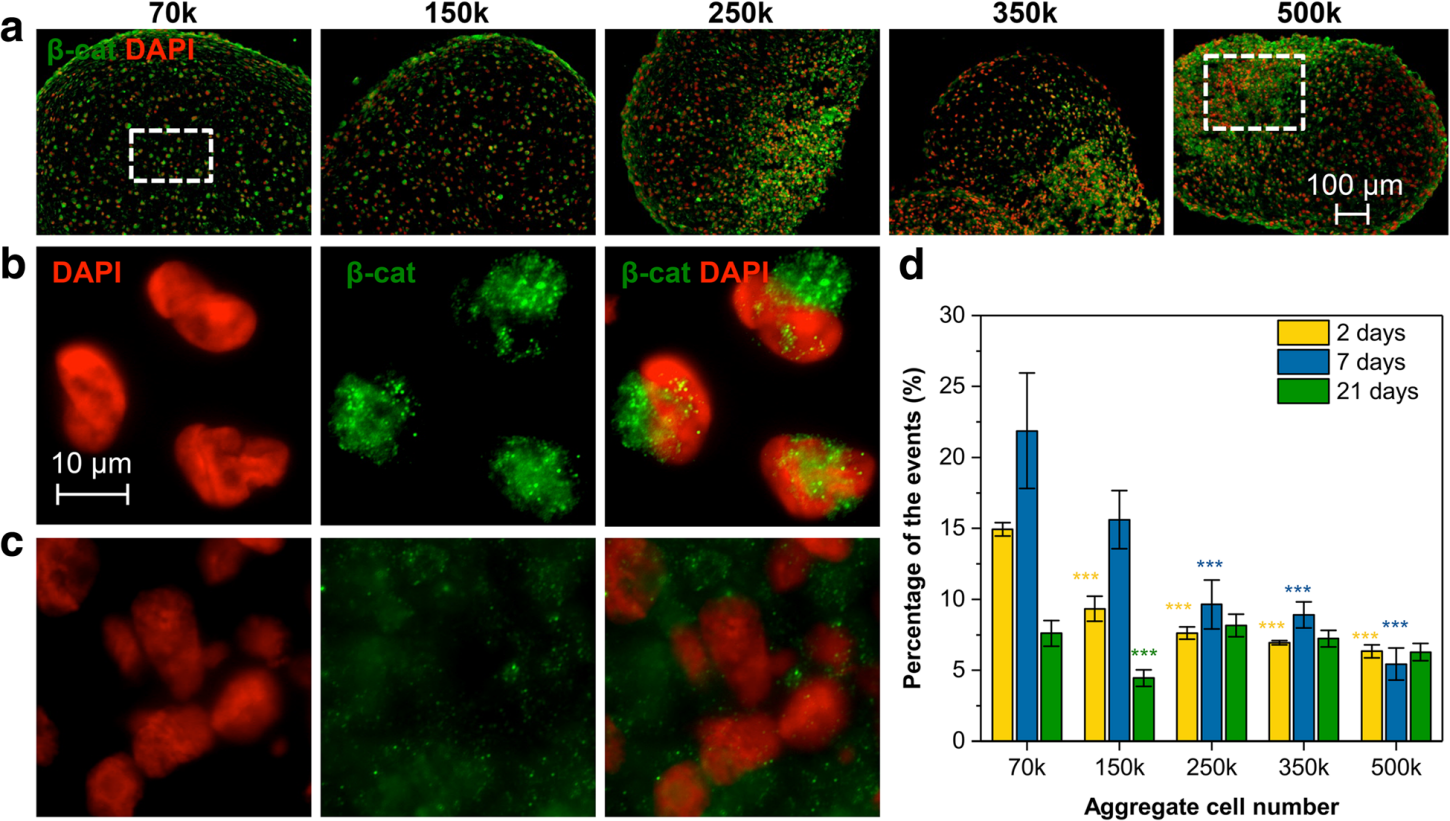
ACN modulates the transcriptional activity of β-catenin. **a** Immunofluorescence staining of β-catenin in sections of pellets after 7 days of differentiation. **b** High magnification images of β-catenin staining in 70 k pellets. **c** High magnification images of β-catenin staining in 500 k pellets. **d** Flow cytometry analysis of ShRNA-transfected MSCs for β-catenin signaling. Values are normalized to GAPDH. Statistical analysis is based on a one-way ANOVA tool of OriginPro. Error bar represents standard deviation, and different aggregates in one group are compared to 70 k in the same group (*p < 0.05, **p < 0.01, ***p < 0.001)

Another important actor in MSC differentiation is β-catenin, a transcriptional co-activator of the canonical Wnt pathway. The role of Wnt family of secreted glycolipoproteins in many embryonic developmental events is well established [56]. It is known that activation of the canonical Wnt pathway is constituent with chondrogenesis [25, 57, 58]. It has been shown that β-catenin expression during limb bud development is higher between days 1 and 4 suggesting a critical role for β-catenin in MSC condensation phase [25, 59]. It has been shown that the extracellular domain of cadherins forms intercellular bonds with surrounding cells through cadherins, while the intracellular domain of cadherins recruits catenins. Recent investigations have shown that cadherin/catenin complexes could participate in the transduction of mechanical forces during development [15]. Interestingly, we saw no major differences in the overall expression of β-catenin between the various conditions (Fig. 5a) baring the general decrease in total β-catenin expression at day 21, which is expected as chondrogenesis reaches homeostasis (Additional file 1: Figure S4). This led us to postulate that, in *low*-ACN condition, a mechanism involving stabilization of β-catenin might be responsible for driving chondrogenesis. The fate of β-catenin in the cytoplasm is dictated by the presence of binding partners that prevent its degradation and N-cad complexation with β-catenin protects it from adenomatous polyposis coli (APC)-axin degradation machinery. Upon stabilization by N-cad and reaching a critical concentration, β-catenin can translocate to the nuclei and bind to the transcription factors of the T cell factor/lymphoid enhancer factor (TCF/LEF) family driving gene expression by switching the target genes from stage of activation to transcription. However, Cav-1 can also interact with β-catenin. It has been shown in epithelial cells that an increase in the expression of Cav-1 leads to the recruitment of the majority of β-catenin to the cell membrane, thereby diminishing the availability of β-catenin for complexation with TCF/LEF and inhibiting β-catenin-mediated transcription activity [60]. Also, it has been shown in fibroblasts that Cav-1 expression can modulate Wnt/β-catenin signaling by regulating the intracellular localization of β-catenin [48]. Thus, the competition between cadherins and Cav-1 for this limited pool of β-catenin can regulate the transcriptional activity of β-catenin [60]. Our finding that N-cad expression is significantly upregulated in *low*-ACN aggregates and that increasing ACN results in upregulation of Cav-1 would imply that in *low*-ACN conditions, β-catenin stabilization in the cytoplasm and shuttling to the nucleus would be favored. In contrast, in *high*-ACN environment, the recruitment β-catenin to the cell membrane would be favored limiting its transcriptional activity. Visualization of the expression pattern of β-catenin after 7 days of differentiation revealed two important differences that lend credence to this conclusion. (1) While β-catenin expression pattern was more uniform in *low*-ACN aggregates, it was heterogeneous in *high*-ACN aggregates (Fig. 6a), and (2) a higher fraction of cells in *low*-ACN aggregates showed localization of β-catenin within the nuclei (Fig. 6b, c). These observations in sum imply that the β-catenin transcription activity in *low*-ACN aggregates should be higher. To investigate this, aggregates were formed using MSCs transfected with commercially available ShRNA (7TGC) to stably express a β-catenin/TCF reporter and then dissociated at different time points and analyzed by flow cytometry for β-catenin transcriptional activity. Transfected MSCs in the absence of nuclear β-catenin expressed only red fluorescence protein; however, upon β-catenin binding to TCF/LEF, green fluorescent protein (GFP) expression is induced [61]. Surprisingly cells in aggregates with lowest ACN showed a threefold increase in the percentage of GFP-positive cells on the second day of differentiation followed by a fivefold increase at day 7 compared to the highest ACN (Fig. 6d). This intriguing finding which correlates well with N-cad expression pattern is consistent with the postulate stated earlier and provides circumspect evidence that in MSCs, in the condensation phase, i.e. up to day 7, the cytoplasmic domains of N-cad interact with β-catenin making stable catenin-cadherin complexes, which then lead to translocation of β-catenin inside the nuclei [62–64]. The absence of such differences in β-catenin transcription activity at day 21 would be consistent with the maturation of the chondrogenesis process as a whole (Fig. 6d).

### ACN impacts expression and activation of MMP-2, a negative regulator of chondrogenesis

The progression of chondrogenesis is accompanied by ECM deposition and remodeling, where matrix metalloproteinases (MMPs) play a critical role in breaking down the ECM leading to release growth factors and unraveling of cryptic binding sites for cells. It has been observed in chick limb bud-derived MSCs that MMP-2 activation during the pre-condensation step inhibits chondrogenesis by negatively regulating cell adhesion [65]. We therefore analyzed the conditioned media from the aggregates at day 7 and day 21 using gelatin zymography and found that secretion of pro-MMP-2 after 7 days increased dramatically (~ 4–5-fold) with increasing ACN, and *high*-ACN aggregates showed activation of MMP-2 while in the other aggregates, there was no obvious sign of MMP-2 activation. However, by day 21, differences between the expressions of MMP-2 between samples were almost abolished but activation of MMP-2 was still 4–5-fold higher in *high*-ACN aggregates (Additional file 1: Figure S5). Considering that increasing ACN negatively impacts chondrogenesis, the direct correlation between Cav-1 expression and expression and activation of MMP-2 with increasing ACN led us to further postulate that upregulation of Cav-1 most likely interferes with MSC condensation during chondrogenic differentiation by impacting ECM remodeling. Interestingly, evidence for an association between Cav-1 and MMP-2 has been found in endothelial cells where it has been shown that MMP-2 co-localizes with Cav-1 on the cell surface and that Cav-1 contains both the proposed receptor and the activator of MMP-2 [66].

### *Low*-ACN promotes expression of fibronectin by MSCs

ECM secreted by cells can have a modulatory role in cell adhesion. FN, a high-molecular-weight glycoprotein, is a key component of the ECM of MSCs, and its expression is upregulated during MSC condensation both in vitro and in vivo [6, 67]. FN possesses cell adhesion domains that play a vital role in mediating cell-cell contact, and it has been suggested that the role of FN in limb bud might be to provide a scaffold for MSCs and promote the formation of pre-cartilage cellular aggregates [6, 68]. FN also supports the deposition of collagen and binding protein for latent TGF-β1 and therefore also has an instructive role in a cartilage matrix formation. WB analysis revealed differences in the expression of fibrillar FN between *low*-ACN and *high*-ACN aggregates starting at day 2, with a three- to fourfold higher expression of FN after 7 days of differentiation in *low*-ACN aggregates compared to *high*-ACN aggregates (Fig. 5a). By day 21, the pronounced difference was absent and FN expression in aggregates with *low*-ACN was markedly diminished suggesting a maturation/homeostasis in chondrogenesis (Additional file 1: Figure S4). Since the insoluble fibrillar form of FN that is present in the ECM is assembled from secreted soluble FN through complex cell-mediated process [69–71], we therefore quantified the amount of soluble FN in the conditioned media of the aggregates at different time points using an in-house developed ELISA (see the “Materials and methods” section for details). We made a compelling finding that the soluble FN in conditioned media from different aggregates throughout the differentiation phase was in a narrow range (11–24 μg/mL) suggesting some autoregulation; however, when normalized to DNA content within the aggregates (i.e., normalization to cell number), FN secretion in early stages of differentiation (day 2 and day 7) in *low*-ACN aggregates was ~ 2–3-fold higher compared to *high*-ACN aggregates (Additional file 1: Figure S6). This finding also correlates with WB analysis of fibrillar FN in the ECM at days 2 and 7 (Fig. 5a). This is the first report showing a direct correlation between the FN secretion capacity of MSC and its potential to undergo chondrogenic differentiation and alludes to the possible use of FN expression as a biomarker for the chondrogenic potential of human MSCs. The FN level in *low*-ACN aggregates also correlates with higher β-catenin transcriptional activity (Fig. 6, Additional file 1: Figure S7), and this conforms to reports in the literature that FN is a direct target of β-catenin signaling [72, 73]. This is also in agreement with our proliferation data (Fig. 3d) as it has been reported that increased FN levels during acinar development result in overproliferation of mammary epithelial cells and acinar size [74]. Interestingly, a recent study has shown that in rat trabecular meshwork cells, stiffness correlated with the expression of FN [75].

Our finding alludes to a complex mechanism involving activation of the canonical Wnt pathway, changes to Cav-1 and FN expression, and cell stiffness in the regulation of chondrogenesis.

### ACN exerts control over MSCs proliferation through regulation of survivin

**Fig. 7:**
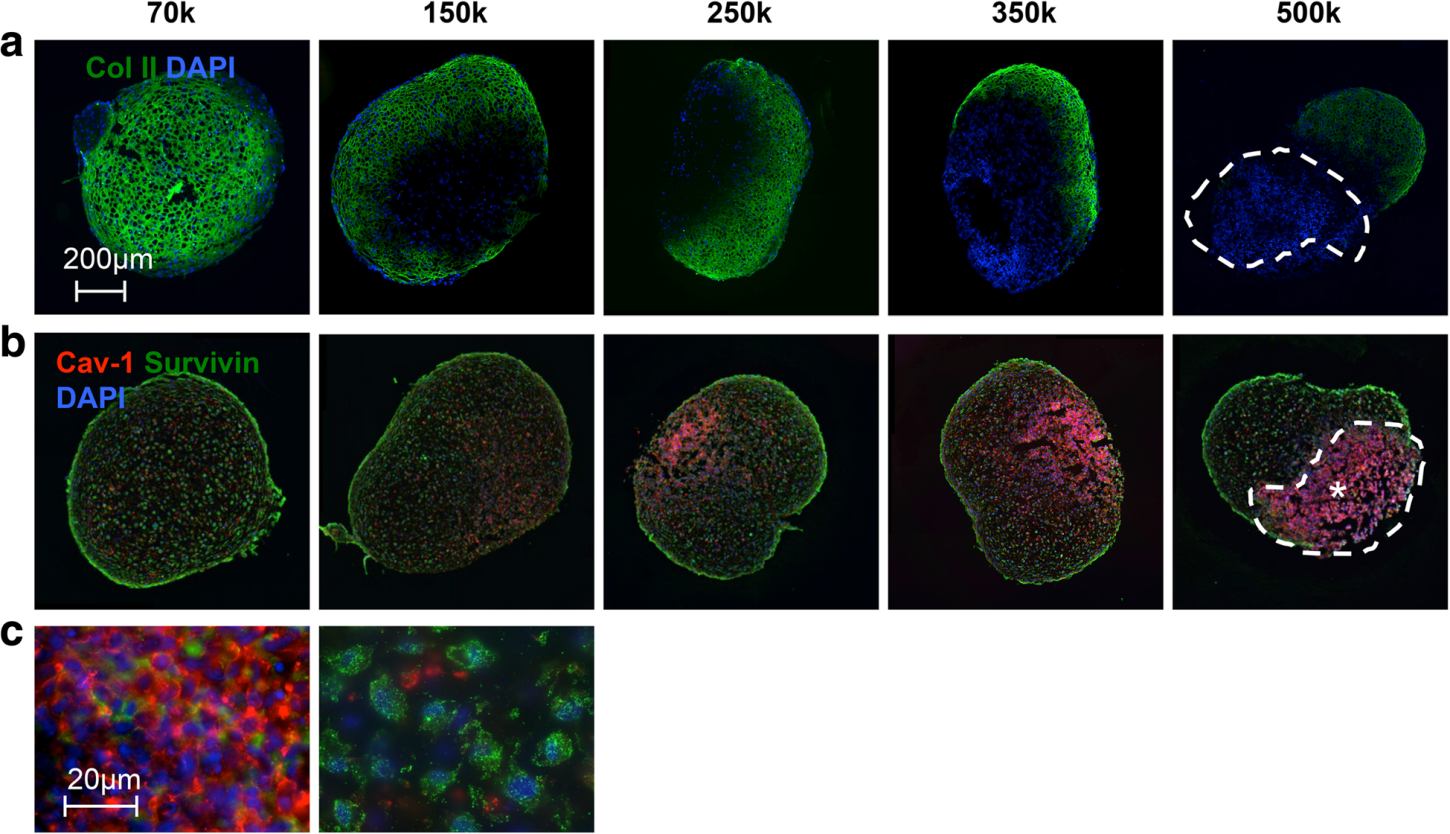
Expression of collagen type II, caveolin-1, and survivin is modulated by ACN. **a** Immunofluorescence staining for collagen type 2 in sections of pellets with different initial cell number after 7 days of differentiation. The area with no expression of collagen type 2 is highlighted by a dashed outline. Scale bar is 200 μm. **b** Immunofluorescence staining for Cav-1 and survivin in sections of pellets with different initial cell number after 7 days of differentiation. The area with high Cav-1 expression is highlighted by a dashed outline. **c** Left: composite of a high magnification z-stack image of Cav-1 and survivin staining in the area donated by asterisk (*) in **b**. Right: focal plane in the middle of the stack

It has been reported in fibroblasts and epithelial cells that Cav-1 expression suppresses cell proliferation by arresting cells in the G_0_/G_1_ phase [76] via the suppression of survivin, a member of the inhibitor of apoptosis proteins [77]. This prompted us to explore the expression of survivin in the context of Cav-1 expression. IF staining of cryosections at day 7 showed that Cav-1 expression was markedly increased with increasing ACN (Fig. 7b), and additionally, regions of low chondrogenicity as indicated by the absence of type-2 collagen expression (Fig. 7a) coincided with regions of high Cav-1 expression. As can be seen in Fig. 7, increasing ACN is accompanied with increasing heterogeneity of Cav-1 expression; this could be rationalized by the fact that MSCs are a heterogeneous population and might respond differently to local microenvironment [78]. Interestingly, the survivin expression pattern showed an inverse relationship with Cav-1 expression pattern (Fig. 7b). High magnification z-stack image from *high*-ACN aggregates (denoted by an asterisk) revealed that the cells with strong expression of Cav-1 lacked expression of survivin and vice versa (Fig. 7c). Our results provide evidence for the first time for an inverse correlation between Cav-1 and survivin expression in human MSCs during the condensation phase. Importantly, this mechanistic relationship between Cav-1, cadherins, and β-catenin transcriptional activity has parallels in the survival mechanisms of metastatic epithelial tumors [79] and provides impetus to extend findings from tumor development and metastasis to skeletal development.

### Toward engineering more efficient constructs for bone and cartilage tissue engineering and regenerative medicine

In the bone and cartilage tissue engineering, acquiring a sufficient number of the cells for in vitro culture or in vivo implantation is costly, time-consuming, and remains the rate-limiting step for clinical translation due to the inverse correlation between MSCs differentiation potential and expansion time. To address this issue, we have investigated if implanting few *low*-ACN in the collagenous matrix will lead to superior outcomes in comparison with traditional methodology of dispersing cells uniformly throughout the matrix. We implanted four 70 k aggregates 2 days post-culture within the collagen matrix and compared it to the outcomes in a collagen matrix seeded with one million MSCs. After 3 weeks of chondrogenic differentiation, the cells within the aggregates were fully mature hypertrophic chondrocytes and were incorporated in the matrix and the deposited tissue was highly homogenous (Additional file 1: Figure S8a). In spite of the total cell number within the aggregate condition being almost four times lower than the conventional culture condition, the outcomes as assessed by the Bern score, which is indicative of the uniformity of the cartilage matrix, was both high and uniform in the aggregate condition (Additional file 1: Figure S8b). Furthermore, interestingly, the developmentally inspired approach inhibited the formation of the hypoxic and necrotic zone in, which occurs due to diminished oxygen and nutrition diffusion in large constructs. This finding validates the premise of our study and demonstrates the framework for potential translational applications.

## Conclusions

Induction of stable chondrogenesis from MSCs is crucial for cartilage tissue engineering, repair of cartilage lesions using MSCs, and bone regeneration using EO paradigms. While significant effort has gone into optimizing culture conditions, serial expansion of cells in vitro for generation of adequate cell numbers for manipulation (tissue engineering and in vivo implantation) comes at the expense of loss in chondrogenic potential. In this study, a donor-independent solution is presented that addressed donor variability and loss of chondrogenic phenotype. We have demonstrated a direct relationship between the cell numbers during MSC aggregation, MSC proliferation, and chondrogenic differentiation. Furthermore, enhanced chondrogenesis correlates with the emergence of a stiffer MSC phenotype, which is accompanied by regulation of proteins involved in mechanotransduction namely Cav-1 and N-cad. Interestingly, a higher expression of survivin, an apoptosis inhibitor, is observed in the low ACN environments. To exalt the translational potential of our findings, in a proof of concept study, it is demonstrated that chondrogenesis that is superior to a conventional approach can be achieved using fourfold less cell numbers by implanting *low*-ACN aggregates in a collagen scaffold. Our analysis showed that the aggregate approach yields superior outcomes by inhibiting the formation of necrotic core and increasing efficiency of matrix deposition. The results of our study provide compelling evidence for a role for cellular mechanics in chondrogenic differentiation of MSCs in 3D aggregates with implication for understanding the mechanisms involved in skeletogenesis and MSC-based regenerative therapies.

## Additional file

Additional file 1: **Table S1.** Average dimensions of the ACN pellets at day 7 of chondrogenic differentiation. **Table S2.** Parameters used in modeling ACNs. **Figure S1.** ACN regulates chondrogenic differentiation of human bone marrow derived MSC. Higher magnification images of Alcian blue staining of pellet sections presented in Fig. 1a and b. **Figure S2.** ACN regulates chondrogenic differentiation of human bone marrow derived MSC. a: Alcian blue staining (low and high magnifications) of pellet sections after 21 days chondrogenic differentiation from 50 years old healthy female (second donor). b: Bern score associated with each MSCs aggregates differentiated in presence of TGF-β1 after 21 days determined from Alcian blue staining of pellets. Statistical analysis is based on One-Way ANOVA tool of Origin-Pro. Error bars are standard error of the mean and different aggregates in one group are compared to 70k in that group. (* *p*<0.05, ** *p*<0.01, *** *p*<0.001). **Figure S3.** ACN regulates progression of human bone marrow derived MSC toward hypertrophy. a: Collagen type 2 and 10 staining of pellet sections after 7 days chondrogenic differentiation. b: Collagen type 2 and 10 staining of pellet sections after 21 days of chondrogenic differentiation. **Figure S4.** ACN effect on proteins regulating condensation is abolished after 21 days of differentiation. Cav-1, N-cad, β-catenin and FN expression doesn’t show appreciable differences between different conditions of ACN. **Figure S5.** Gelatin zymography and quantification of conditioned media from aggregates at day 7 and day 21. a: Gelatin zymography of conditioned media from aggregates in different time points. b: Quantification of pro MMP-2 at day 7 of chondrogenesis. c: Quantification of active MMP-2 at day 21 of chondrogenesis. **Figure S6.** Quantification of soluble fibronectin (FN) in conditioned media using ELISA. Data is normalized to cell number and expressed as concentration on a per cell basis per day. **Figure S7.** Correlation between concentration of soluble fibronectin (FN) in conditioned media (measured using ELISA) wit activation of b-catenin signaling (measured using FACS) in early stages of chondrogenesis in different aggregates. **Figure S8.** a:Safranin-O staining of tissue generated using 4×70k MSCs aggregate (denoted by dashed black circles) in comparison to control (1 million MSCs seeded in collagen foam). Cells within the aggregates show progression toward hypertrophic chondrocyte and the generated tissue shows homogenous deposition of proteoglycan. Additionally, by employing low-ACN aggregates the formation of hypoxic core (denoted by white arrow) is inhibited. b: Histomorphometric analysis of generated tissue in both constructs. (PDF 17344 kb)

## Abbreviations

ACN: Aggregate cell number
ALPL: Alkaline phosphatase
ASPN: Asporin
ASS1: Argininosuccinate synthase 1
BDKRB1: Bradykinin receptor B1
BMP4: Bone morphogenetic protein 4
C3: Complement component 3
CAV1: Caveolin-1
Col: Collagen
DKK1: Dickkopf-related protein 1
DMEM: Dulbecco’s modified essential medium
ECM: Extracellular matrix
EO: Endochondral ossification
FN: Fibronectin
FZD4: Frizzled class receptor 4
GAG: Glycosaminoglycans
GFP: Green fluorescent protein
HES1: hes family bHLH transcription factor 1
HIF: Hypoxia-inducible factor
HILPDA: Hypoxia-inducible lipid droplet-associated
HMGB2: High mobility group box 2
IBSP: Integrin-binding sialoprotein
IGF: Insulin-like growth factor
IHH: Indian hedgehog
IRAK2: Interleukin-1 receptor-associated kinase 2
ITGB3: Integrin beta 3
KLF9: Kruppel-like factor 9
LEP: Leptin
MEM: Minimum essential medium
MSCs: Mesenchymal stems cells
N-cad: N-cadherin
NEAA: Non-essential amino acids
NFKBIA: Nuclear factor of kappa light polypeptide gene enhancer in B cell inhibitor
NR1D1: Nuclear receptor subfamily 1, group D, member 1
NR4A3: Nuclear receptor subfamily 4, group A, member 3
OCT: Optimal cutting temperature
PANX3: Pannexin 3
PDE: Partial differential equation
PDE4D: Phosphodiesterase 4D
PTH1R: Parathyroid hormone-1 receptor
RT-DC: Real-time deformability cytometry
SIK1: Salt-inducible kinase 1
TGF-β1: Transforming growth factor-beta 1
TNFRSF1B: Tumor necrosis factor receptor superfamily, member 1B
