# Supplementary material for: Cell number in mesenchymal stem cell aggregates dictates cell stiffness and chondrogenesis
Source: Stem Cell Res Ther. 2019 Jan 10;10:10. doi: 10.1186/s13287-018-1103-y (PMC6329065; doi:10.1186/s13287-018-1103-y)
Supplement: Supplementary file 1 — Table S1. Average dimensions of the ACN pellets at day 7 of chondrogenic differentiation. Table S2. Parameters used in modeling ACNs. Figure S1. ACN regulates chondrogenic differentiation of human bone marrow derived MSC. Higher magnification images of Alcian blue staining of pellet sections presented in Fig. 1a and b. Figure S2. ACN regulates chondrogenic differentiation of human bone marrow derived MSC. a: Alcian blue staining (low and high magnifications) of pellet sections after 21 days chondrogenic differentiation from 50 years old healthy female (second donor). b: Bern score associated with each MSCs aggregates differentiated in presence of TGF-β1 after 21 days determined from Alcian blue staining of pellets. Statistical analysis is based on One-Way ANOVA tool of Origin-Pro. Error bars are standard error of the mean and different aggregates in one group are compared to 70k in that group. (* p<0.05, ** p<0.01, *** p<0.001). Figure S3. ACN regulates progression of human bone marrow derived MSC toward hypertrophy. a: Collagen type 2 and 10 staining of pellet sections after 7 days chondrogenic differentiation. b: Collagen type 2 and 10 staining of pellet sections after 21 days of chondrogenic differentiation. Figure S4. ACN effect on proteins regulating condensation is abolished after 21 days of differentiation. Cav-1, N-cad, β-catenin and FN expression doesn’t show appreciable differences between different conditions of ACN. Figure S5. Gelatin zymography and quantification of conditioned media from aggregates at day 7 and day 21. a: Gelatin zymography of conditioned media from aggregates in different time points. b: Quantification of pro MMP-2 at day 7 of chondrogenesis. c: Quantification of active MMP-2 at day 21 of chondrogenesis. Figure S6. Quantification of soluble fibronectin (FN) in conditioned media using ELISA. Data is normalized to cell number and expressed as concentration on a per cell basis per day. Figure S7. Correlation between concentration of solu [file 13287_2018_1103_MOESM1_ESM.pdf]

Supplementary Table. 1

ACN	Radius (μm)	Volume (μm^3)	Cell density ($1/\mu m^3$)
**70k**	429.41	3.3167e+08	2.1105e-04
**150k**	453.22	3.8996e+08	3.8466e-04
**250k**	518.1	5.8254e+08	4.2915e-04
**350k**	569.69	7.7447e+08	4.5192e-04
**500k**	504.95	5.3930e+08	9.2712e-04

Supplementary Table 1: Average dimensions of the ACN pellets at day 7 of chondrogenic differentiation.

Supplementary Table. 2

**Oxygen**	Diffusion coefficient around 2e-9 [m2/s][1, 2]
	update rate 12 fmol/cell/h [3] in normoxia
	concentration at normoxia about 7.46 mol/m^3
**TGF-β1**	diffusion coefficient 2.13e-11 [m2/s]
	uptake rate: 3.5275e-18 [g/cell/min]
	concentration in medium: 10 [ng/ml]
**Glucose**	Diffusion coefficient 5e-10 [m^2/s]
	Molecular weight 180.156 [g/mol]
	Uptake rate: 210 [fmol/cell/h] = 3.78e-11 [g/cell/h] [3]
	Concentration in medium: 4.5 [mg/ml]

Supplementary Table 2: Parameters used in modeling ACNs.

Supplementary Figure. 1

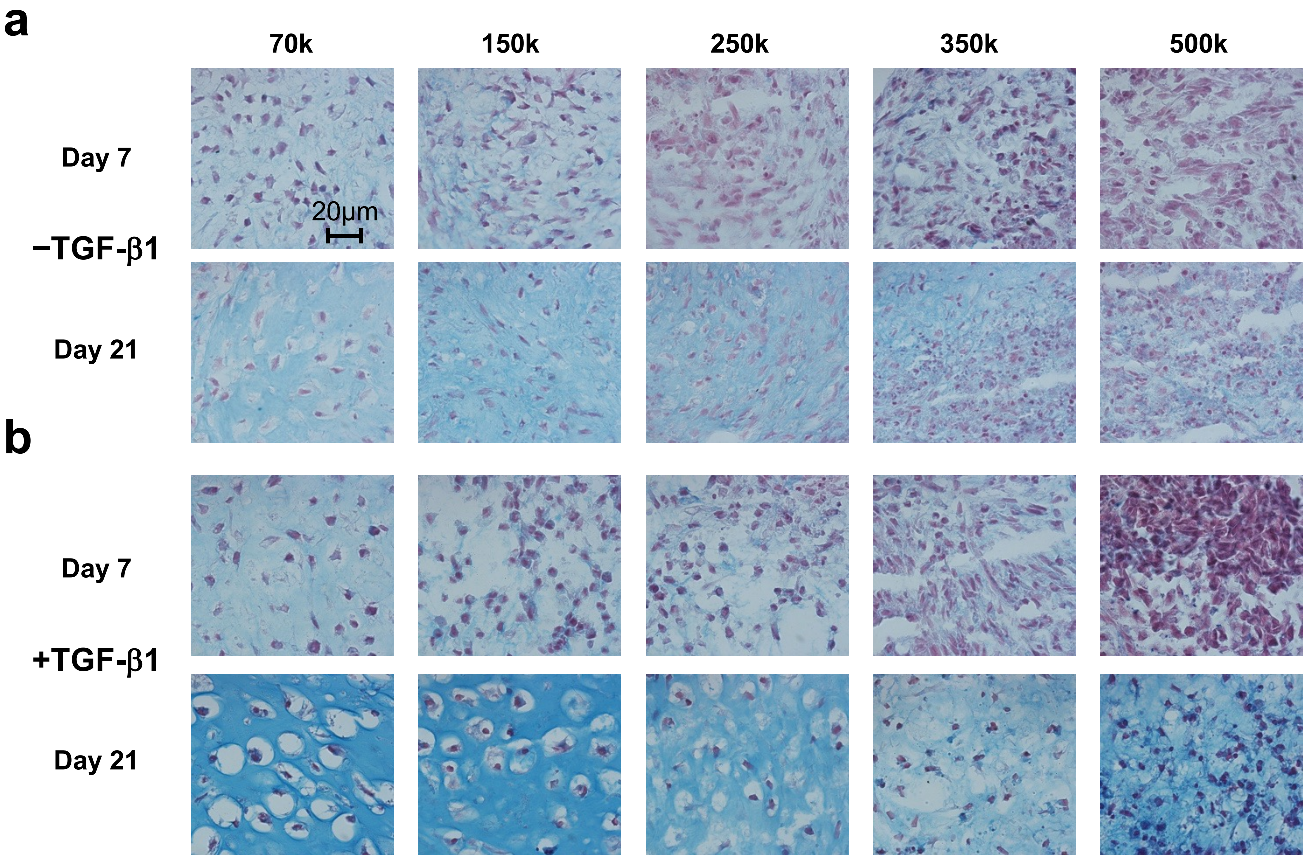

Supplementary Figure 1: ACN regulates chondrogenic differentiation of human bone marrow derived MSC. Higher magnification images of Alcian blue staining of pellet sections presented in Figure 1 a and b.

Supplementary Figure. 2

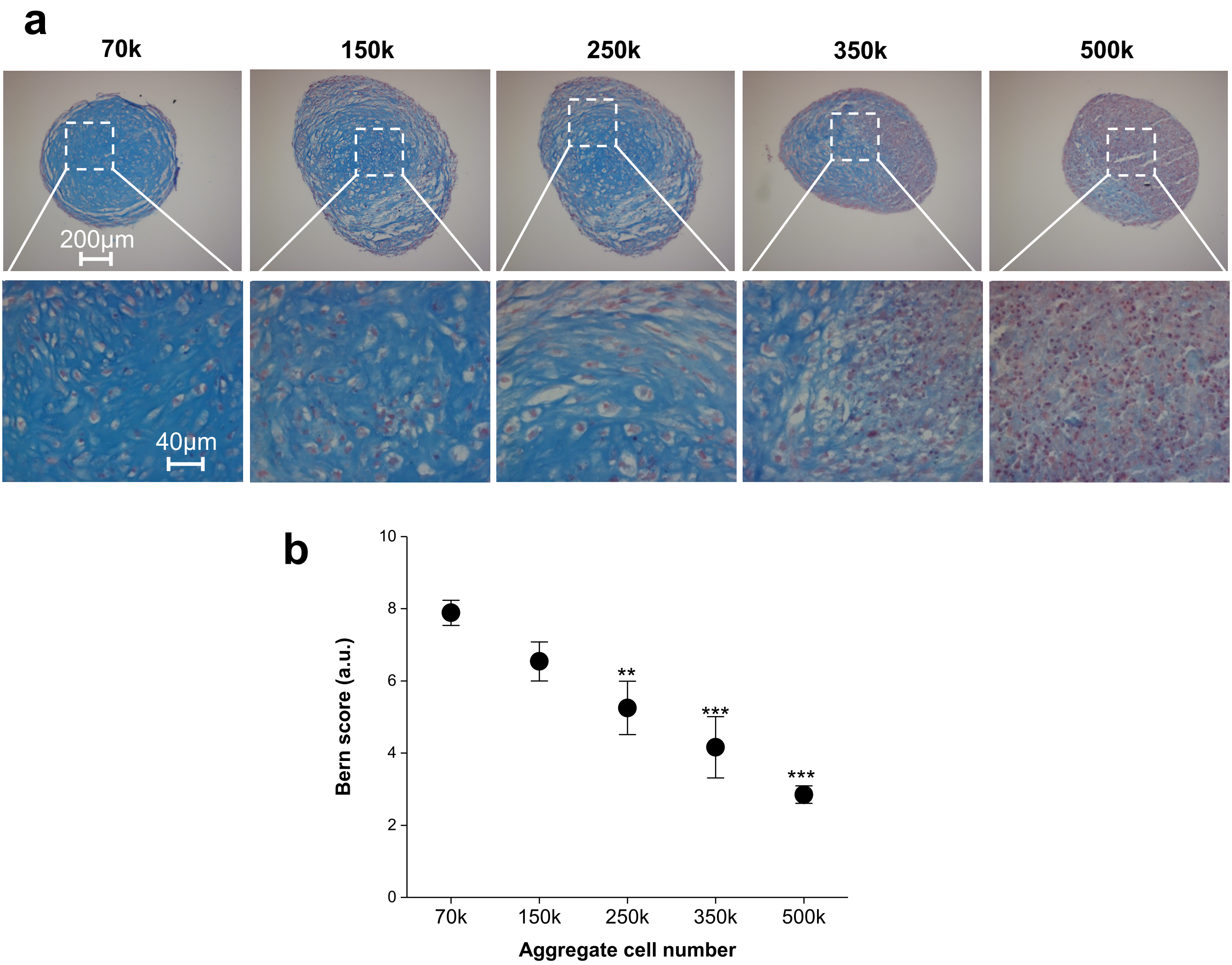

Supplementary Figure 2: ACN regulates chondrogenic differentiation of human bone marrow derived MSC. **a:** Alcian blue staining (low and high magnifications) of pellet sections after 21 days chondrogenic differentiation from 50 years old healthy female (second donor). **b**: Bern score associated with each MSCs aggregates differentiated in presence of TGF-β1 after 21 days determined from Alcian blue staining of pellets. Statistical analysis is based on One-Way ANOVA tool of Origin-Pro. Error bars are standard error of the mean and different aggregates in one group are compared to 70k in that group.(* $p<0.05$, ** $p<0.01$, *** $p<0.001$)

Supplementary Figure. 3

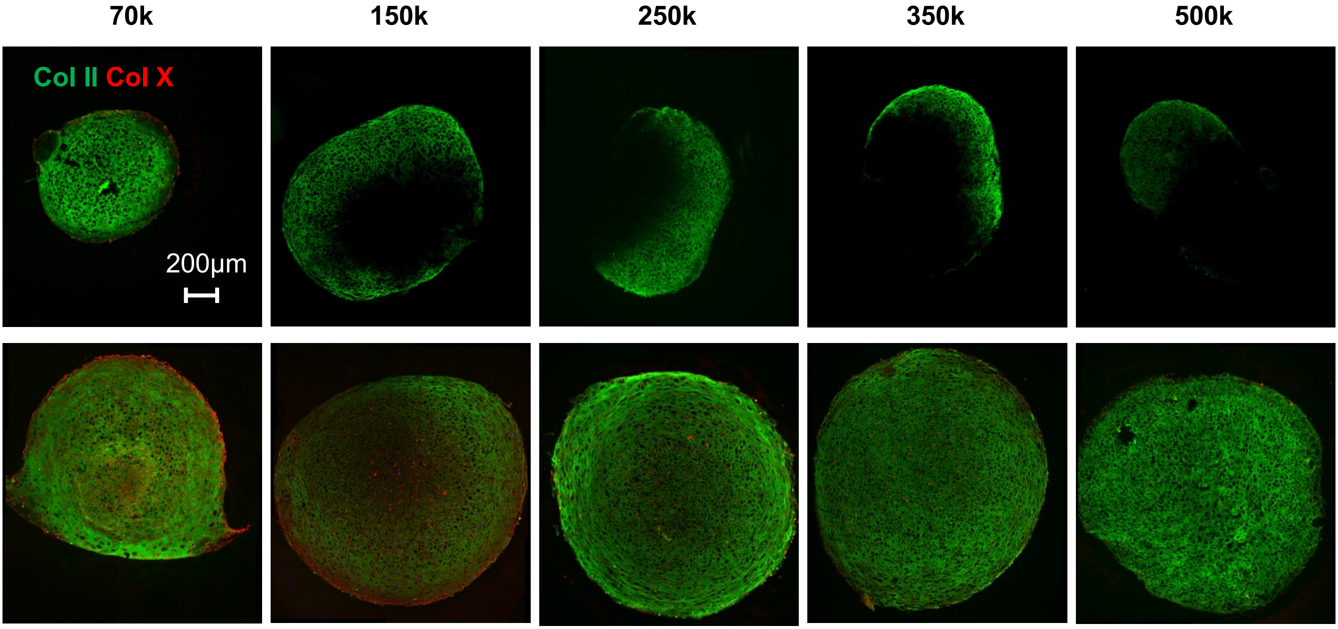

Supplementary Figure 3: ACN regulates progression of human bone marrow derived MSC toward hypertrophy (a) Collagen type 2 and 10 staining of pellet sections after 7 days chondrogenic differentiation. (b) Collagen type 2 and 10 staining of pellet sections after 21 days of chondrogenic differentiation.

Supplementary Figure. 4

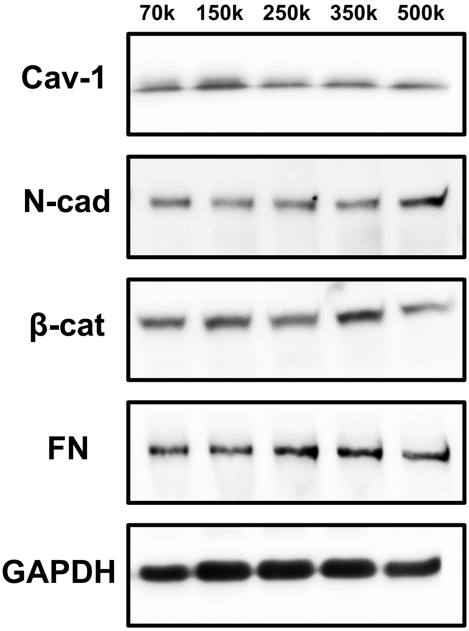

Supplementary Figure 4: ACN effect on proteins regulating condensation is abolished after 21 days of differentiation. Cav-1, N-cad, β-catenin and FN expression doesn't show appreciable differences between different conditions of ACN.

Supplementary Figure. 5

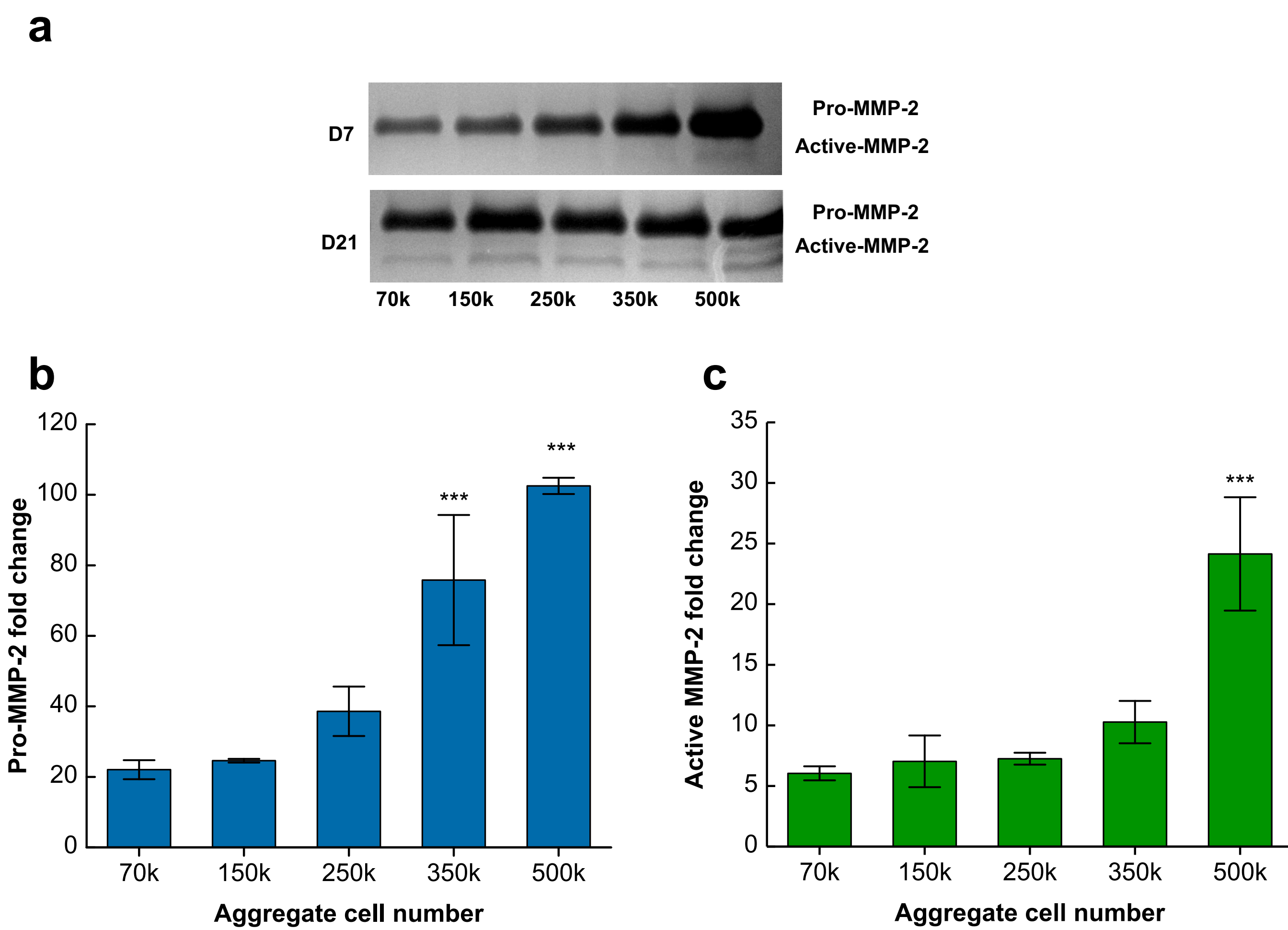

Supplementary Figure 5: Gelatin zymography and quantification of conditioned media from aggregates at day 7 and day 21. **a**: Gelatin zymography of conditioned media from aggregates in different time points. **b**: Quantification of pro MMP-2 at day 7 of chondrogenesis. **c**: Quantification of active MMP-2 at day 21 of chondrogenesis.

Supplementary Figure. 6

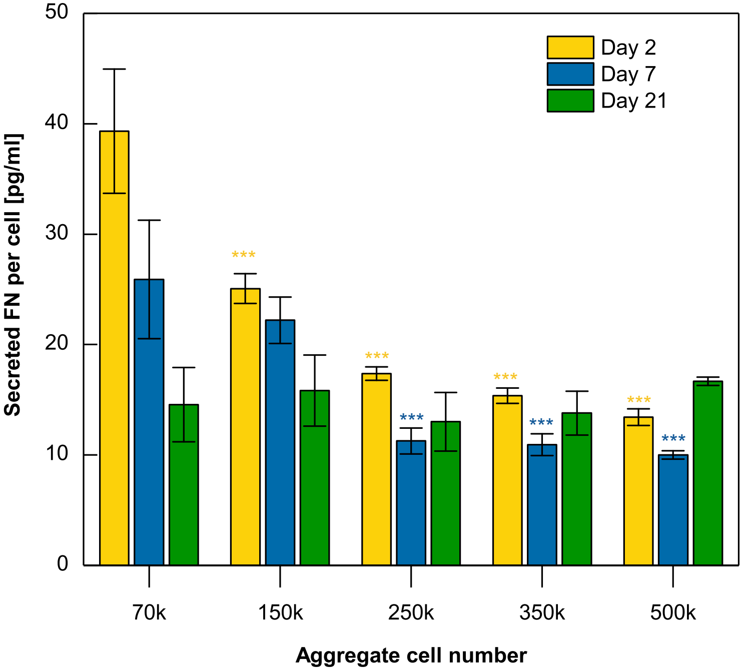

Supplementary Figure 6: Quantification of soluble fibronectin (FN) in conditioned media using ELISA. Data is normalized to cell number and expressed as concentration on a per cell basis per day.

Supplementary Figure.7

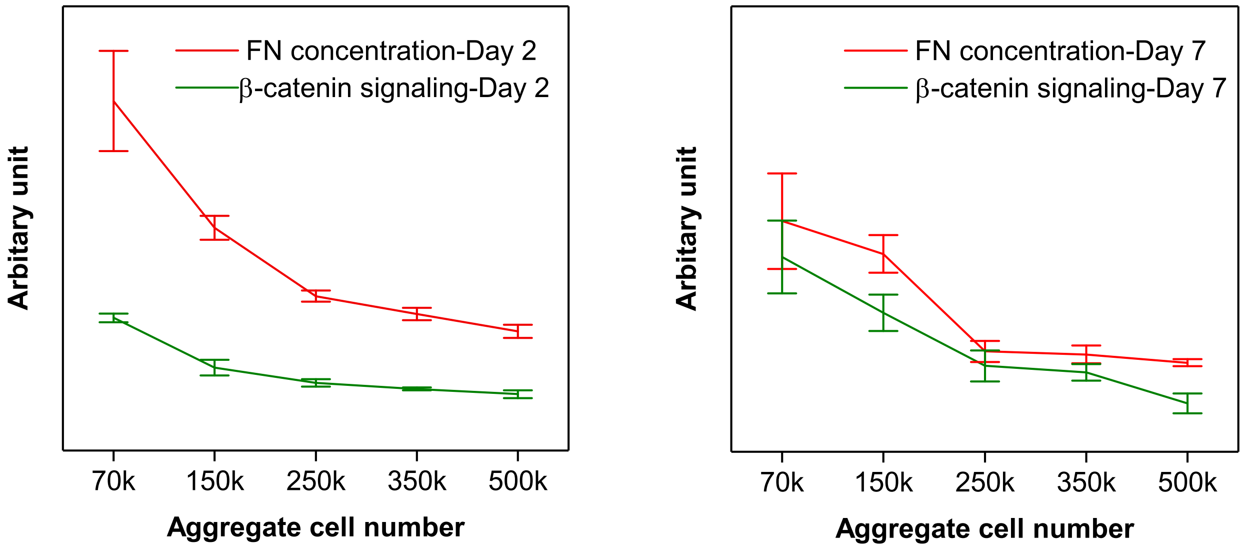

Supplementary Figure 7: Correlation between concentration of soluble fibronectin (FN) in conditioned media (measured using ELISA) wit activation of β-catenin signaling (measured using FACS) in early stages of chondrogenesis in different aggregates.

Supplementary Figure. 8

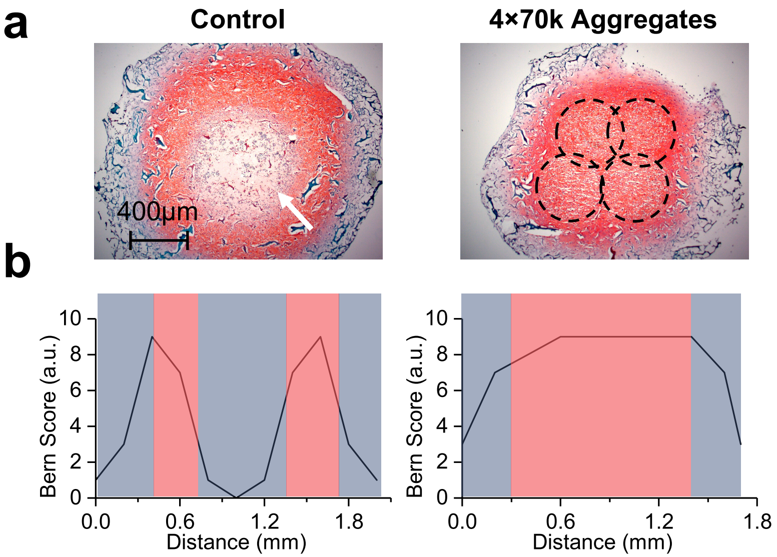

Supplementary Figure 8: a: Safranin-O staining of tissue generated using 4×70k MSCs aggregate (denoted by dashed black circles) in comparison to control (1 million MSCs seeded in collagen foam). Cells within the aggregates show progression toward hypertrophic chondrocyte and the generated tissue shows homogenous deposition of proteoglycan. Additionally, by employing low-ACN aggregates the formation of hypoxic core (denoted by white arrow) is inhibited. **b**: Histomorphometric analysis of generated tissue in both constructs.

References:

1. Oxygen diffusion through natural extracellular matrices: implications for Estimating "critical thickness" values in tendon tissue engineering. Tissue Engineering, 2008.

2. Determination of the effective diffusion coefficient of oxygen in gel materials in relation to gel concentration. Biotechnology Techniques, 1989.

3. The metabolism of human mesenchymal stem cells during proliferation And differenttaion. Cellular Physiology, 2010.8

4. Vilar, Jose MG, Ronald Jansen, and Chris Sander. "Signal processing in the TGF-β superfamily ligand-receptor network." PLoS computational biology 2.1 (2006): e3.

5. Chung, Seung-Wook, et al. "Quantitative modeling and analysis of the transforming growth factor β signaling pathway." Biophysical journal 96.5 (2009): 1733-1750.
